# Anti-Inflammatory Activity of Fruit Fractions *in Vitro*, Mediated through Toll-Like Receptor 4 and 2 in the Context of Inflammatory Bowel Disease

**DOI:** 10.3390/nu6115265

**Published:** 2014-11-19

**Authors:** Noha Ahmed Nasef, Sunali Mehta, Pamela Murray, Gareth Marlow, Lynnette R. Ferguson

**Affiliations:** Faculty of Medical & Health Sciences, University of Auckland, Auckland 1023, New Zealand; E-Mails: n.nasef@auckland.ac.nz (N.A.N.); s.mehta@auckland.ac.nz (S.M.); P.Murray@auckland.ac.nz (P.M.); g.marlow@auckland.ac.nz (G.M.)

**Keywords:** Toll-Like Receptors, inflammatory bowel disease, polyphenols, inflammation

## Abstract

Pattern recognition receptors such as Toll-Like Receptor 2 (TLR2) and 4 (TLR4) are important in detecting and responding to stress and bacterial stimuli. Defect or damage in the TLR2 and TLR4 pathways can lead to sustained inflammation, characteristic of inflammatory bowel disease (IBD). The goal of this study was to identify fruit fractions that can be tested further to develop them as complementary therapies for IBD. In order to do this, we identified fruit fractions that mediate their anti-inflammatory response through the TLR4 and TLR2 pathway. Human Embryonic Kidney (HEK)-hTLR4 and hTLR2 cells were stimulated with their respective ligands to induce inflammation. These cells were treated with one of the 12 fractionated fruits and the inflammatory effect measured. 10 of the fruits came up as anti-inflammatory in the hTLR4 assay and nine in the hTLR2 assays. Many of the fruit fractions mediated their anti-inflammatory actions either mainly in their hydrophobic fractions (such as elderberry) or hydrophilic fractions (such as red raspberry), or both. The strongest anti-inflammatory effects were seen for feijoa and blackberry. This study shows that fruits can have multiple fractions eliciting anti-inflammatory effects in a pathway specific manner. This suggests that the compounds found in fruits can act together to produce health benefits by way of reducing inflammation. Exploiting this property of fruits can help develop complimentary therapies for inflammatory diseases.

## 1. Introduction

The gastrointestinal tract uses a system of tolerance and controlled inflammation to limit the response of bacteria-derived antigens in the gut resulting in mutual benefit for both the host and the host microbiota [1]. When this complex system breaks down by a pathogenic insult in a genetically predisposed individual, the resulting immune response may lead to inflammatory bowel disease (IBD) [1].

In normal conditions, when pattern recognition receptors (PRRs) on the surface of cells are triggered by noxious conditions such as bacterial antigens, acute inflammation is activated to remove the triggering insult [2]. A successful acute inflammatory response results in the elimination of the infectious agent followed by resolution of inflammation and tissue repair [3]. However, in abnormal conditions such as IBD, prolonged inflammation can occur leading to increased tissue damage in the inflamed area [4].

Prolonged inflammation may also lead to epithelial cell necrosis (caused by excessive inflammation and tissue injury) and the subsequent release of damage-associated molecular pattern molecules (DAMPs) [5]. DAMPs have the ability to activate PRRs such as Toll-like Receptors 2 and 4 (TLR2 and TLR4) [6,7], and in turn induce further secondary inflammation in a repeating cycle that ultimately results in self-sustaining chronic inflammation [8]. DAMPs in combination with increased sensitivity to bacteria [9,10,11] can result in the over activation of PRRs and lead to chronic inflammation.

The primary focus for treating IBD disease is to reduce the flare-ups in the bowel when the disease is active and to keep inflammation at normal levels during time of remission [12]. Unfortunately the treatments that are currently available for IBD have limited success and are not effective on some patients, while others have significant side effects [13,14]. Limited success of treatment suggests the need for complementary intervention. One such method includes dietary intervention to help improve the management of IBD.

Evidence suggests that fruits have a wide variety of plant polyphenols that have anti-inflammatory properties and are increasingly regarded as effective protective agents against disease [15,16,17,18,19,20,21,22]. Therefore, to identify fruits that are relevant to study in relation to TLRs and chronic inflammatory diseases, we have used an *in vitro* screen to study the ability of hydrophilic and hydrophobic extracts from 12 fruits to modulate the inflammatory response by activation of PRRs including TLR4, TLR2/1 and TLR2/6. In this study, we show that several fruit extracts from strawberry, blackberry and feijoa have the ability to mediate an anti-inflammatory effect, when inflammation is stimulated through PRRs. Results from our study suggest that these fruits or fruit extracts can be further developed to be used as a complementary intervention to improve the management of IBD patient treatment.

## 2. Experimental Section

### 2.1. Cell Line and Culture Medium

Engineered Human Embryonic Kidney (HEK) cell line HEK-blue hTLR2 and HEK-blue hTLR4 (Invivogen, San Diego, CA, USA) were maintained in high glucose Dulbecco’s Modified Eagle’s Medium (DMEM) containing sodium pyruvate and l-glutamine (Life technologies, Carlsbad, CA, USA) supplemented with 10% fetal bovine serum (FBS) (life technologies, Carlsbad, CA, USA), 0.4% HEK-blue selection media (Invivogen, San Diego, CA, USA), 0.2% Normocin (Invivogen, San Diego, CA, USA) and 1% penicillin streptomycin (PSG) (Life Technologies, Carlsbad, CA, USA). HEK-blue hTLR4 and hTLR2 cells can only be stimulated through activation of the TLR4 or TLR2 receptors respectively. These cells have been stably transfected with Secreted Embryonic Alkaline Phosphatase (SEAP) plasmid containing NFKβ response elements. The cells were maintained at 37 °C in a humidified 5% carbon dioxide (CO_2_) incubator. All experiments were conducted using DMEM containing sodium pyruvate and l-glutamine supplemented with 10% FBS. When the NFKβ (marker of inflammation) is stimulated with a TLR4 lipopolysaccharides (LPS) or TLR2 ligand (Pam3CysSerLys4 (PAM3CSK4) and Pam2CGDPKHPKSF (FSL-1)) it will result in the expression of SEAP that can be detected using a colorimetric assay [23].

### 2.2. Fruits

Twelve fruits were selected for screening based on their polyphenol content and evidence of their anti-inflammatory properties (Table 1). Selection criteria for each of these fruits are listed in Table 1.

**Table 1:** Fruits selected for screening and the criteria used to select these fruits.

Fruits Screened	Selection Criteria	Reference
Mangosteen	Anti-inflammatory and anti-oxidant properties, high xanthone content	[24,25,26,27]
Feijoa	Anti-microbial and anti-oxidant properties	[28]
Elderberry	High plant polyphenol content	[29]
Cranberry	Chemo-preventative properties including anti-inflammatory effects	[30]
Blackcurrant	High plant polyphenol content	[29]
Blackberry	High plant polyphenol content	[29]
Red raspberry	High plant polyphenol content	[29]
Strawberry	High plant polyphenol content	[29]
Green Grapes	High plant polyphenol content	[29]
Plum	High plant polyphenol content	[29]
Pear	High plant polyphenol content	[29]
Black Grapes	High plant polyphenol content	[29]

#### Fractionation

All fruit fractions were kindly provided by Nigel Perry with help from Elaine Burgess and Wendy Smith from Plant and Food Research (Ruakura, New Zealand). About 1 kg of fresh fruit was acquired and the normally consumed parts of the fruit, including the skin were freeze-dried and ground up. 10 g of the fruit was homogenized in 100 mL of ethanol then mixed overnight on a shaker at room temperature and filtered under vacuum on the following day. 50 mL of the filtered solution was coated onto 6 g of octadecyl-functionalized silica gel (C18; Sigma Aldrich, St. Louis, MO, USA) and dried using a rotary evaporator in a water bath at 30 °C. Next, the dried bulk mix was added to a preconditioned (100% ethanol followed by 50% ethanol followed by water) 25 mL, 5 g, C18 isolute separation phase extraction (SPE) cartridge. Two hydrophilic fractions (F1 and F2) were collected from the cartridge by eluting in 100% water. Fractions with decreasing hydrophilic strengths including F3 and F4, F5 and F6, F7 and F8 were collected from the cartridge by eluting in 75% water and 25% ethanol, 50% water and 50% ethanol, 25% water and 75% ethanol, respectively. Finally hydrophobic fractions F9 and F10 were collected from the cartridge by eluting in 100% ethanol. Additional hydrophobic fractions F11 and F12 were collected from the cartridge by eluting in 100% ethanol acetate. The fractions 1 to 8 were transferred to a speed vacuum concentrator (CentriVap, labconco, Kansas, USA), for drying overnight at ambient temperature. The dried fractions 1 to 8 were re-dissolved in ethanol or a mix of ethanol and water for the more water-soluble fractions. The re-dissolved fractions were aliquoted out and then dried again using a speed vacuum concentrator. Fractions 9 to 12 were dried using a Nitrogen BlowDown Dry Evaporator and re-dissolved in 100% ethanol. The dissolved fractions were then aliquoted out and left to dry in the speed vacuum concentrator. All fractions were stored at −20 °C until required. Each aliquot contained the equivalent of 1 g of whole food. Each aliquot was sterilized using gamma irradiation at the standard dose of 25–32 Kilogray (Schering-Plough Animal Health Limited, Wellington, New Zealand). The sterilized samples were then dissolved in 1 mL of 20% Dimethyl sulfoxide (DMSO, Sigma-Aldrich, St. Louis, MO, USA) and 80% autoclaved Milli-Q water. Dissolved fractions 2 to 11 were then studied using *in vitro* assays. Fractions 1 and 12 were not used for the *in vitro* assays since they may have contained non-specific products eluting at the beginning and end of each fractionation run.

### 2.3. HEK-Blue Anti-Inflammatory Screening of Fruit Fractions

#### Anti-Inflammatory Screening in HEK-Blue Cell Line

HEK-blue hTLR2 and hTLR4 cells were seeded into 96 well plates at a cell density of 4 × 10^5^ cells/mL in DMEM containing sodium pyruvate and l-glutamine supplemented with 10% FBS. The plates were then incubated overnight at 37 °C, 5% CO_2_ incubator. Fruit fractions 2 to 11 were made up in DMEM containing sodium pyruvate and l-glutamine supplemented with 10% FBS. The top concentration of the fruit fractions used for the TLR4 was 1:8 of each fraction (equivalent to 17.8 mg of whole food) and for the TLR2 assay was 1:60 of each fraction (equivalent to 2.4 mg of whole food). This was then serially diluted 2-fold to make up a total of 4 dilutions of each fraction. A lower dose for the fruit fractions was used because the HEK-blue hTLR2 cell line gave cytotoxic results at the doses used for the TLR4 screen.

Controls included 100 ng/mL ultrapure LPS (TLR4 ligand) to ensure no hTLR4 cells contaminated the hTLR2 assay, 100 ng/mL PAM3CSK4 (TLR2 ligand) to ensure no hTLR2 cells contaminated the hTLR4 assay, 0.25 μg/mL Phorbol myristate acetate (PMA; NFKβ stimulator) and media. Solvent control contained 20% DMSO and 80% autoclaved Milli-Q water. 20 μL of either the fractions or controls was added into each well 48 h after cell seeding.

The plates were then placed back in the incubator for an hour. Following the incubation, a further 20 μL of either media or one of three TLR ligands were added. The ligand added was either 3.125 μg/mL LPS (Ultrapure, Invivogen, San Diego, CA, USA) to stimulate TLR4, 10 ng/mL PAM3CSK4 (Invivogen, San Diego, CA, USA) which stimulated TLR2/TLR1, or 10 ng/mL Pam2CGDPKHPKSF (FSL-1) (Invivogen, San Diego, CA, USA) which stimulated TLR2/TLR6. Then, the plates were incubated for a further 4 h.

To measure the expression of NFKβ, Quanti-blue (QB, Invivogen, San Diego, CA, USA) was used as per the manufacturer’s instructions. At the end of the 4 h incubation, 40 μL of the cell supernatant was transferred from the cell plate into a new plate containing 100 μL per well QB and left incubating at room temperature for 1 h. After the hour all QB plates were read using a plate reader (Multiskan, Thermo Labsystems, Waltham, USA) at a test wavelength of 650. The anti-inflammatory activity score for each fruit fraction was calculated by subtracting the QB score for the solvent from the QB score of the individual fractions. The equation is summarized as follows:

(1) Anti-inflammatory activity score = Solvent control QB score − Fraction QB score

To measure cytotoxicity induced by the ligand, 2-(4-iodophenyl)-3-(4-nitrophenyl)-5-(2,4-disulfophenyl)-2H-tetrazolium, monosodium salt (WST-1) (Roche, Basel, Switzerland) was used according to the manufacturer instructions. WST-1 is a reagent used for the measurement of cellular proliferation, viability, and cytotoxicity using a colorimetric assay. 20 μL of WST-1 was added into the cell plate after the supernatant for QB was removed and further incubated for an hour at 37 °C. Inflammation increased the cell’s metabolic activity which resulted in a high WST-1 score. If a fraction is anti-inflammatory, the cytotoxicity score goes down. In order to avoid the confounding effect of ligand induced inflammation on the WST-1 score, media without ligand was used as a reference. A WST-1 score below 80% was considered cytotoxic. The cytotoxicity score was based on the WST-1 measurement and was calculated as follows:

(2) [WST-1 score for each sample/WST-1 score for the media control (no ligand)] × 100

## 3. Results

### 3.1. HEK Screening Results

**Figure 1:**
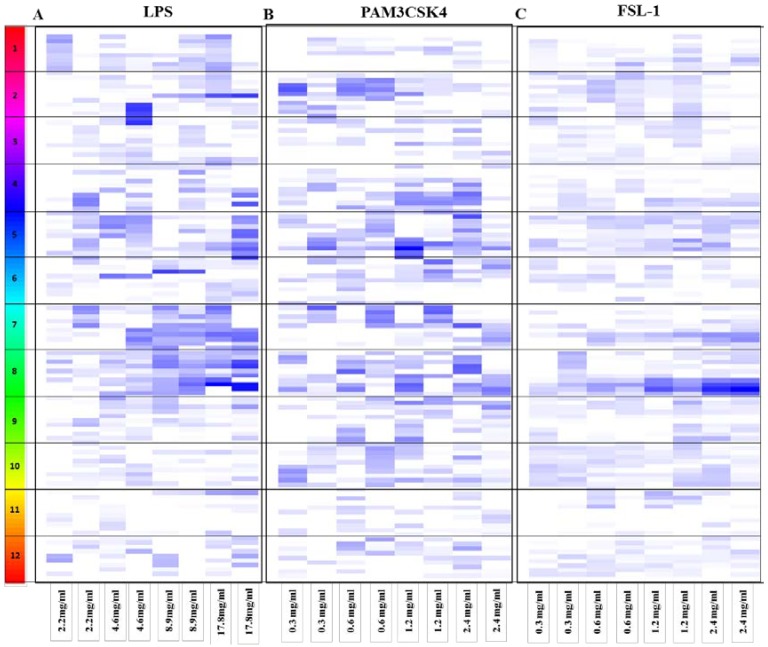
Anti-inflammatory activity score for the individual fruit fractions. Each fruit is represented by a number on the left legend: 1-Pear, 2-Elderberry, 3-Blackcurrant, 4-Red raspberry, 5-Blackberry, 6-Cranberry, 7-Strawberry, 8-Feijoa, 9-Black grapes, 10-Green grapes, 11-Mangosteen and 12-Plum. For each fruit, each row represents a fruit fraction with fraction 11 (hydrophobic) from the top to fraction 2 (hydrophilic) going downwards. Each column represents a concentration. For each concentration there are two biological replicates. The white indicates the fraction was either cytotoxic (Equation (2)) hence excluded from further analyses or had no anti-inflammatory effect. Scale of blue represents the anti-inflammatory score (Equation (1)), light blue (weak anti-inflammatory score) to dark blue (strong anti-inflammatory score). Details of the cytotoxicity scores and anti-inflammatory scores are provided in the supplementary data. (**A**) HEK-Blue hTLR4 (LPS); (**B**) HEK-Blue hTLR2, TLR2/TLR1 (PAM3CSK4) and (**C**) HEK-Blue hTLR2, TLR2/TLR6 (FSL-1) screening. LPS, lipopolysaccharides; PAM3CSK4, Pam3CysSerLys4; FSL-1, Pam2CGDPKHPKSF.

The anti-inflammatory activity of the fractionated fruits was tested by analyzing inhibition of TLR mediated NFKβ activation in response to stimulation by either LPS (TLR4 specific ligand), PAM3CSK4 (TLR2/1 specific ligand), or FSL-1 (TLR2/6 specific ligand), in HEK-Blue hTLR4 and hTLR2 cells respectively. TLR induction of NFKβ was quantitated for each fruit fraction and was represented using the anti-inflammatory activity score (Equation (1)), illustrated in Figure 1.

Anti-inflammatory activity scores varied for the individual fruit fractions tested using the HEK-Blue hTLR4 cells stimulated by LPS (TLR4 specific ligand). In addition, several fractions showed cytotoxicity at the highest concentration of 17.8 mg/mL and the anti-inflammatory scores were excluded from the results shown in Figure 1. Details of the cytotoxicity scores and anti-inflammatory scores are provided in the supplementary data. Results from the HEK-Blue hTLR4 screen suggest that green grapes, pear, feijoa, black grapes, elderberry, blackcurrant, mangosteen, strawberry, blackberry and cranberry mediate an anti-inflammatory effect. Of these fruits, the feijoa fractions showed the most prominent effects. Overall, there were no obvious anti-inflammatory trends towards either the hydrophilic fractions (F2 to F6) or the hydrophobic fractions (F7 to F11) (Figure 1). However some fruits, including pear, blackcurrant, strawberry and blackberry mediated their anti-inflammatory effect mainly through their hydrophilic fractions. On the other hand, plum, feijoa, black grapes, mangosteen, elderberry and cranberry mediated their anti-inflammatory effect via their hydrophobic fractions.

Similarly, anti-inflammatory activity scores varied for the individual fruit fractions tested using the HEK-Blue hTLR2 cells stimulated by PAM3CSK4 (TLR2/1 specific ligand). Results from this screening suggested that feijoa, black grapes, blackberry, raspberry, elderberry, cranberry and green grapes mediate their anti-inflammatory effect in this pathway. Overall, in this screen ~70% of the fractions, mediating an anti-inflammatory effect via the TLR2/1 pathway were hydrophilic. Of these fruits, the feijoa fractions showed the most prominent effects, especially in F2 to F5. Other fruits that mainly mediated their anti-inflammatory effect through their hydrophilic fractions included plum, red raspberry and strawberry. On the other hand, elderberry had a weak anti-inflammatory score in its hydrophobic fractions F8 and F9.

In contrast, less anti-inflammatory activity was observed by the individual fruit fractions tested using the HEK-Blue hTLR2 cells stimulated by FSL-1 (TLR2/6 specific ligand). Results from this screen suggest that elderberry, green grapes, plum, blackberry, red raspberry, cranberry, black grapes, strawberry and feijoa fruits mediate their anti-inflammatory effect via the TLR2/6 pathway. Similar to the TLR4 results, there were no obvious anti-inflammatory trends towards either the hydrophilic fractions (F2 to F6) or the hydrophobic fractions (F7 to F11). However, black grapes, and cranberry mediated their anti-inflammatory effect mainly through the hydrophobic fractions, while blackberry, red raspberry and green grapes showed only anti-inflammatory effects in their hydrophilic fractions.

Results from the *in vitro* screens suggest that some fruit fractions work via specific pathways, while others are more ubiquitous in nature. Fruit fractions that mediate their anti-inflammatory effect specifically via the TLR2/1 and TLR4 pathway include F9 of green grapes, F4 of feijoa and F8 of elderberry. On the other hand, F5 of blackberry and red raspberry mediated their anti-inflammatory effect via the TLR2 and TLR4 pathways.

### 3.2. Dose Response for the Anti-Inflammatory Extracts

From the preliminary analyses of the *in vitro* screens (Figure 1), we selected a number of fruits whose fractions (hydrophilic and hydrophobic) mediated an anti-inflammatory response upon stimulation with LPS (TLR4 specific ligand), PAM3CSK4 (TLR2/1 specific ligand) or FSL-1 (TLR2/6 specific ligand) based on two criteria. Firstly, fruit fractions were chosen when both biological replicates mediated an anti-inflammatory effect in their respective screens. Secondly, where there was an anti-inflammatory trend in response to an increase in the concentration of the individual fruit fraction.

In the hTLR4 LPS screen, the strongest anti-inflammatory response exhibited by hydrophilic fractions was mediated by feijoa and in particular feijoa F4 (Table 2). Feijoa F4 showed a gradual increase in the anti-inflammatory effect starting with no response at the lowest dose of 2.2 mg/mL, and increasing to a score of 0.15 ± 0.008 at the highest dose of 17.8 mg/mL (Table 2). On the other hand feijoa F6 (0.06 ± 0.031) and blackcurrant F3 (0.038 ± 0.023) showed a less pronounced anti-inflammatory response at the highest dose of 17.8 mg/mL.

**Table 2:** Anti-inflammatory response mediated by hydrophilic (A–C) and hydrophobic (D–F) fractions. Concentration columns showing two replicates for each fraction. LPS, lipopolysaccharides; PAM3CSK4, Pam3CysSerLys4; FSL-1, Pam2CGDPKHPKSF.

Treated	Fruit & Fraction	Concentration (mg/mL)							
		2.2		4.4		8.9		17.8	
A. LPS	Feijoa F4	−0.03	0.00	0.03	0.02	0.05	0.09	0.16	0.15
	Feijoa F6	−0.01	0.02	−0.03	0.02	0.03	0.05	0.04	0.08
	Black currant F3	−0.01	0.00	0.01	0.03	0.03	0.02	0.05	0.02
		**0.3**		**0.6**		**1.2**		**1.4**	
B. PAM3CSK4	Green grapes F3	0.03	0.05	0.00	0.01	0.01	0.04	0.05	0.01
	Green grapes F4	0.03	0.02	0.03	0.02	0.02	0.04	0.05	0.01
	Blackberry F4	0.03	0.04	0.01	0.04	0.04	0.09	0.05	0.04
	Red raspberry F4	0.00	0.03	0.02	−0.02	0.05	0.03	0.05	0.05
	Red raspberry F5	0.00	0.03	0.03	0.00	0.06	0.05	0.03	0.03
	Strawberry F4	0.00	−0.02	0.07	0.03	0.04	0.03	0.08	0.1
	Strawberry F5	0.01	0.04	0.05	0.04	0.06	0.08	0.1	0.1
	Feijoa F2	0.05	0.02	0.05	0.03	0.08	0.04	0.14	0.15
	Feijoa F3	0.04	0.04	0.09	0.04	0.16	0.12	0.20	0.23
	Feijoa F4	0.03	0.06	0.09	0.09	0.12	0.09	0.17	0.17
	Feijoa F5	0.01	0.12	0.05	0.08	0.12	0.04	0.11	0.04
		**0.3**		**0.6**		**1.2**		**1.4**	
C. FSL–1	Blackberry F4	0.03	0.08	0.03	0.04	0.13	0.04	0.04	0.06
	Blackberry F5	0.00	0.06	0.01	0.02	0.08	0.07	0.05	0.02
	Red raspberry F5	0.01	−0.02	0.00	0.02	0.06	0.05	0.06	0.02
	Green grapes F3	0.06	0.00	0.01	0.03	0.04	0.03	0.04	0.02
		**2.2**		**4.4**		**8.9**		**17.8**	
D. LPS	Feijoa F8	0.01	0.01	0.03	0.04	0.08	0.06	0.08	0.12
	Feijoa F9	0.05	−0.01	0.04	0.01	0.08	0.05	0.06	0.11
	Feijoa F10	0.01	−0.01	0.02	0.02	0.07	0.03	0.05	0.03
	Mangosteen F10	0.00	−0.01	−0.01	0.00	0.02	0.02	0.06	0.06
	Elderberry F8	−0.01	−0.01	0.00	0.00	0.05	0.06	0.11	0.12
		**0.3**		**0.6**		**1.2**		**1.4**	
E. PAM3CSK4	Elderberry F8	0.02	0.01	0.04	0.02	0.01	0.02	0.01	0.01
	Elderberry F10	0.03	0.03	0.05	0.02	0.02	0.02	0.01	−0.01
	Blackberry F10	0.02	0.03	0.04	0.04	0.06	0.02	0.05	0.06
	Blackberry F11	0.05	0.01	0.06	0.04	0.05	0.04	0.05	0.04
		**0.3**		**0.6**		**1.2**		**1.4**	
F. FSL–1	Black grapes F10	0.00	−0.01	0.01	0.02	0.06	0.05	0.04	0.04
	Cranberry F9	0.00	−0.01	0.02	0.00	0.02	0.06	0.03	0.02

The hTLR2 (PAM3CSK4) screen showed a number of hydrophilic fractions with strong anti-inflammatory properties (Table 2). Similar to the LPS screen, feijoa fractions had the strongest anti-inflammatory effect. Feijoa F3 had the strongest anti-inflammatory score starting with 0.04 ± 0.0 at the lowest feijoa F3 concentration of 0.3 mg/mL and increasing to a score of 0.22 ± 0.03 at the highest concentration of 2.4 mg/mL. Feijoa F4 (0.17 ± 0.0 at 2.4 mg/mL; 0.05 ± 0.03 at 0.3 mg/mL) also showed a strong anti-inflammatory effect similar to what was seen in the LPS screen. This was followed by feijoa F2 (0.15 ± 0.0 at 2.4 mg/mL; 0.03 ± 0.02 at 0.3 mg/mL) and feijoa F5 (0.08 ± 0.06 at 2.4 mg/mL; 0.06 ± 0.08 at 0.3 mg/mL) (Table 2). Next came strawberry F5 starting with a weak response of 0.03 ± 0.02 at the lowest concentration (0.3 mg/mL) and increasing to a score of 0.1 ± 0.0 at the highest concentration (2.4 mg/mL); followed by strawberry F4 with no effect at the lowest concentration and increasing to an anti-inflammatory score of 0.09 ± 0.02 at the highest concentration. Other hydrophilic fractions showing an anti-inflammatory response in this screen includes red raspberry F4 (0.05 ± 0.0 at 2.4 mg/mL) and F5 (0.03 ± 0.0 at 2.4 mg/mL), blackberry F4 (0.04 ± 0.01 at 2.4 mg/mL), green grapes F3 (0.03 ± 0.03 at 2.4 mg/mL) and F4 (0.03 ± 0.03 at 2.4 mg/mL).

In the hTLR2 (FSL-1) screen, there were only very weak anti-inflammatory effects mediated through this pathway by hydrophilic fractions (Table 2). This included red raspberry F5 (0.04 ± 0.03 at 2.4 mg/mL; no response at 0.3 mg/mL), blackberry F4 (0.05 ± 0.02 at 2.4 mg/mL; 0.05 ± 0.04 at 0.3 mg/mL) and F5 (0.04 ± 0.02 at 2.4 mg/mL; 0.03 ± 0.03 at 0.3 mg/mL) and green grapes F3 (0.03 ± 0.02 at 2.4 mg/mL; 0.03 ± 0.04 at 0.3 mg/mL).

In the hTLR4 LPS screen, the strongest anti-inflammatory response by hydrophobic fractions was also mediated by feijoa fractions (Table 2). This included feijoa F8 that gave a score of 0.1 ± 0.03 at 17.8 mg/mL and showed no effect at 2.2 mg/mL; and F9 which scored 0.08 ± 0.04 at 17.8 mg/mL and 0.04 ± 0.02 at 2.2 mg/mL. In addition, elderberry also showed a strong score of 0.1 ± 0.0 at the highest concentration of 17.8 mg/mL, similar to feijoa F8 and F9. Mangosteen F10 showed the lowest anti-inflammatory score of 0.06 ± 0.0 at 17.8 mg/mL.

In contrast to the hydrophilic fractions, the hTLR2 (PAM3CSK4) screen showed very few anti-inflammatory hydrophobic fractions (Table 2). The highest anti-inflammatory scores was for blackberry F10 (0.05 ± 0.0 at 2.4 mg/mL) and F11 (0.04 ± 0.0 at 2.4 mg/mL). Elderberry F8 (0.02 ± 0.0 at 1.2 mg/mL) and F10 (0.02 ± 0.0 at 1.2 mg/mL) showed a weak effect at the lowest concentrations.

There were even less hydrophobic fractions that came up as anti-inflammatory in the hTLR2 (FSL-1) screen (Table 2). Both black grapes F10 (0.04 ± 0.0 at 2.4 mg/mL) and cranberry F9 (0.02 ± 0.0 at 2.4 mg/mL) were weakly anti-inflammatory.

## 4. Discussion

PRRs are found on many cell types and play an important role in activation of inflammatory signals which can result in either a full blown immune response or tolerance [31]. In this study we carried out an *in vitro* screen to identify fractions from 12 fruits capable of mediating an anti-inflammatory effect through the PRRs, TLR2 and TLR4.

Of the 12 fruits tested in this study ten of these fruits were identified in the hTLR4 screen as anti-inflammatory and nine fruits were identified in the hTLR2 screens. Of these, blackberry, feijoa, green grapes, elderberry and cranberry showed up as anti-inflammatory in all the *in vitro* screens suggesting that the compounds they are composed of are capable of interacting with the TLR4 receptors and mediating the anti-inflammatory effect via either the classical MyD88 dependent or the MyD88 independent pathways [32].

One example includes feijoa, which is known to contain large amounts of polyphenols such as, catechin, leucoanthocyanins, flavonols, ellagic acid pentoside, quercitin, hyperin and proanthocynadins [33,34]. A previous study by Rossi *et al.* [35] that examined the anti-inflammatory effect of feijoa’s acetone fractions in a mouse macrophage cell line found that the acetone extracts have anti-inflammatory activity due, in part, to the suppression of nitric oxide production by flavone when the cells were stimulated with LPS.

We found a similar anti-inflammatory effect from the hydrophobic feijoa fractions (F7–F9) in the hTLR4 screen as demonstrated in the mouse macrophage cell line. It is possible that the anti-inflammatory effects seen in feijoa F7–F9 in our study are due to the presence of flavones. Additionally, the study also identified the anti-oxidant action of their feijoa fraction is not a direct effect but is mediated through inhibitory effects on NFKβ and MAP Kinase [35]. Our findings support this result, which suggests that the fruit is involved in the regulation of oxidative stress and inflammation. Similar to feijoa, other fruits like blackberry, green grapes, elderberry and cranberry can also mediate their anti-inflammatory effect by inhibiting NFKβ and MAP Kinase.

Since blackberry, feijoa, green grapes, elderberry, cranberry and plum showed up as anti-inflammatory, it suggests that they can also mediate their anti-inflammatory effect via the TLR2 receptor. TLR2 has been shown to stimulate a parallel pathway apart from the classical MyD88-dependent pathway used by most TLRs. TLR2 is able to stimulate the PI3K-Akt pathway which was shown to attenuate the MAPK-NFKβ-signaling cascade [36]. This pathway is not stimulated by LPS. These findings might explain an alternative mechanism of the anti-inflammatory effect mediated by these fruits in the TLR2 screen at comparatively smaller doses. It is likely that there are several compounds acting specifically in the fruit fractions, which act directly on the MyD88 pathway and indirectly through the PI3K-pathway, which further amplify the inhibition of NFKβ expression.

In terms of absorption in the intestine, one of the factors that is important in absorption of polyphenols is that they must be soluble in the bulk aqueous phase of the gastrointestinal milieu. This would facilitate diffusion through the unstirred water layer that protects the epithelial surface layer [37]. This makes it important to understand the polarity of the fractions tested as this emphasizes that while *in vitro* we are able to see an anti-inflammatory effect; this might not be the case in a physiological setting.

In order to give a better chance of hits in the assay, fruits were fractionated using reverse phase chromatography, to concentrate potential active molecules. This procedure isolates compounds that are likely to be taken up by the cell. In order for polyphenols to be absorbed by the cell, they need to have low molecular weight and have low polarity [38]. Fractionation is therefore a useful method to extract molecules of medium polarity and relatively low molecular weight (<1000 Da) making them more likely to be bioavailable in the assays, excluding the proteins or polysaccharides.

We found several fruits including feijoa, blackberry and green grapes that mediated anti-inflammatory effects via hydrophilic and hydrophobic fractions (Figure 1). These results suggest there is a higher likelihood of these fruits to mediate an anti-inflammatory effect in a physiological setting. These results also indicate that there are multiple active compounds that are having an effect in the fruit. There is also the likelihood that within one fraction there is more than one active compound working together in synergy. Therefore looking at the fractions instead of the compounds is more likely to give a stronger effect.

Some of the fruits screened including elderberry and cranberry mainly had an anti-inflammatory effect in their hydrophobic fractions. These fruits may fail to mediate an anti-inflammatory effect in a physiological setting even though they are positive in our screen and hence need to be followed up carefully. Also for these fruits there may be specific compound(s) that elicit an anti-inflammatory response benefit, thus in this instance it may be worth isolating the active compound rather than use the entire fraction to see a stronger effect.

Elderberry, blackcurrant, blackberry, red raspberry, strawberry, green grapes, plum and pear were selected due to their high polyphenolic content [29]. Despite their high polyphenol content, these fruits did not stand out nor demonstrate a relatively high anti-inflammatory activity score in any of the screens in our study (Figure 1 and Table 2). There are several reasons for this contradictory finding. One of these reasons includes the possibility that the polyphenolic molecules within these fruits, don’t interact with the TLR4 and the TLR2 pathway at the tested concentrations.

Another reason for the limited anti-inflammatory effect mediated by these fruits could be due to the use of the compounds in their native form. For example it has been shown that the total polyphenol content of elderberry increased when it is converted to the aglycone form from the native compound. Also it has been demonstrated that the aglyone form has stronger anti-oxidant levels in a screen using the folin test [29]. Additionally, there could be differences in the cell types used between the different studies. Moreover, differences in the extraction method including the type of solvents used can alter the composition of the fruit fractions, thus altering the results observed. Finally, the content of the fruits might vary from one fruit to another depending on ripeness, season, time of picking and storage.

For the experiments reported in this study, all fruits including feijoa and mangosteen, were fractionated with the skin and pulp. In feijoa, the skin has more bioactivity than the pulp [33]. Some Asian cultures eat the skin however, in most western cultures many people find the taste too sour. Furthermore, mangosteen doesn’t get consumed with the skin. It can therefore be argued that identifying and isolating individual compound(s) that elicit an anti-inflammatory effect would allow for a more controlled administration especially in cases of treatment and management of certain diseases. On the other hand, if the consumption of the fruit were simply for the maintenance of good health, then the natural variability in the fruit would be preferred in order to get the full range of benefits from the whole food.

The purpose of these screens was to identify potentially interesting fruits for further studies in the context of TLR chronic inflammation. As such, this screen was performed as a high throughput, low-resolution screen. Secondly, in these experiments, the assumption is made that the anti-inflammatory effect is mediated by the native compounds. However, the observed health benefit of phytochemicals may not necessarily occur due to the native form that is found in food [39], but by alterations to the native form of the food by the various metabolic processes that occur before or after absorption. These metabolic processes are performed by the digestive enzymes and the gut microflora, that breakdown the phytochemicals into simpler compounds and alter the functional groups of the phytochemical. Therefore the metabolites may actually be the active compound responsible for the biological activity. However, many studies including ours, measure the biological activity of the native phytochemical. Reasons for doing this include, firstly, most phytochemicals can be converted into many metabolites, which exponentially increases the number of metabolites that need to be measured. Secondly, in many situations, the metabolites that are generated from a phytochemical are unknown or incomplete. Thirdly, the activity of the native phytochemical is better characterized than its metabolites both *in vivo* and *in vitro.* Finally, the native compound serves as a marker for all its metabolites even if not a complete one [39].

The HEK-Blue cells were engineered cells from Invivogen and therefore don’t generate a response based on any physiological outcome. Further studies would be ideal to validate the results from this study using primary intestinal cell lines from IBD patients and finally human trials.

## 5. Conclusions

One of the goals of IBD therapies is to reduce inflammation. Therefore identifying anti-inflammatory dietary sources would be ideal in managing inflammatory diseases such as IBD. In this study we used a high throughput screen to identify fruit fractions that have an anti-inflammatory effect. Twelve fruits were screened with varying results. From this study some useful candidates have been identified and in particular feijoa. Further studies into the involvement of these candidates in the PRR pathway can lead to better understanding of how these fruit can help personalize nutrition for better management of disease in the future.

## Supplementary Files

Supplementary File 1
