# Supplementary material for: Anti-Inflammatory Activity of Fruit Fractions in Vitro, Mediated through Toll-Like Receptor 4 and 2 in the Context of Inflammatory Bowel Disease
Source: Nutrients. 2014 Nov 19;6(11):5265–79. doi: 10.3390/nu6115265 (PMC4245588; doi:10.3390/nu6115265)
Supplement: Supplementary File 1 [file nutrients-06-05265-s001.docx]

Supplementary Information

**Table S1.** HRK-blue hTLR4 lipopolysaccharides (LPS) screening scores for 12 fruits. Quanti-blue (QB) score represents [Solvent control QB score] − [Fraction QB score]. The monosodium salt (WST-1) score represents [WST-1 score for each sample/WST-1 score for the media control (no ligand) *] × 100 which is a percentage.

| **Fruit** | **Concentration (mg/mL)** | **Fraction** | **QB (Replicate 1)** | **QB (Replicate 2)** | **WST-1 (%) (Replicate1)** | **WST-1 (%) (Replicate 2)** |
| --- | --- | --- | --- | --- | --- | --- |
| **Feijoa** | **17.8** | F2 | 0.128 | 0.131 | 61.3 | 75.8 |
|  |  | F3 | 0.122 | 0.145 | 71.2 | 90.8 |
|  |  | F4 | 0.156 | 0.145 | 80.4 | 86.4 |
|  |  | F5 | 0.096 | 0.115 | 82.4 | 76.9 |
|  |  | F6 | 0.039 | 0.083 | 87.7 | 117.5 |
|  |  | F7 | 0.009 | 0.04 | 88.8 | 97 |
|  |  | F8 | 0.075 | 0.121 | 95.1 | 118.1 |
|  |  | F9 | 0.059 | 0.108 | 101.1 | 87 |
|  |  | F10 | 0.045 | 0.032 | 84.0 | 86.4 |
|  |  | F11 | 0.039 | 0.051 | 95.5 | 94 |
|  | **8.9** | F2 | 0.055 | 0.067 | 81.0 | 84.8 |
|  |  | F3 | 0.05 | 0.072 | 84.7 | 89.7 |
|  |  | F4 | 0.05 | 0.092 | 85.4 | 88.3 |
|  |  | F5 | 0.048 | 0.075 | 80.7 | 93.7 |
|  |  | F6 | 0.026 | 0.052 | 84.3 | 97.3 |
|  |  | F7 | 0.029 | 0.039 | 104.1 | 101.0 |
|  |  | F8 | 0.078 | 0.059 | 107.2 | 110.7 |
|  |  | F9 | 0.077 | 0.047 | 97.1 | 107.1 |
|  |  | F10 | 0.068 | 0.031 | 92.9 | 95.5 |
|  |  | F11 | 0.046 | 0.035 | 91.7 | 104.0 |
|  | **4.6** | F2 | 0.049 | 0.059 | 81.7 | 91.0 |
|  |  | F3 | −0.003 | 0.03 | 101.8 | 88.7 |
|  |  | F4 | 0.03 | 0.021 | 95.7 | 99.0 |
|  |  | F5 | 0.024 | 0.038 | 90.0 | 105.2 |
|  |  | F6 | −0.025 | 0.022 | 111.1 | 105.5 |
|  |  | F7 | 0.016 | 0.007 | 98.3 | 101.8 |
|  |  | F8 | 0.025 | 0.04 | 100.3 | 104.5 |
|  |  | F9 | 0.044 | 0.014 | 90.0 | 107.3 |
|  |  | F10 | 0.018 | 0.024 | 95.0 | 83.3 |
|  |  | F11 | 0.032 | 0.026 | 91.5 | 96.6 |
|  | **2.2** | F2 | −0.003 | −0.012 | 83.0 | 87.1 |
|  |  | F3 | −0.018 | 0.009 | 91.3 | 110.9 |
|  |  | F4 | −0.025 | −0.001 | 93.2 | 92.1 |
|  |  | F5 | 0.025 | 0.002 | 93.4 | 83.6 |
|  |  | F6 | −0.014 | 0.016 | 97.6 | 100.7 |
|  |  | F7 | 0.034 | 0.016 | 89.4 | 95.6 |
|  |  | F8 | 0.005 | 0.005 | 90.6 | 92.6 |
|  |  | F9 | 0.045 | −0.012 | 92.5 | 100.7 |
|  |  | F10 | 0.008 | −0.013 | 92.6 | 90.1 |
|  |  | F11 | 0.026 | 0.027 | 90.9 | 93.3 |

**Table S1.** *Cont.*

| **Blackberry** | **17.8** | F2 | 0.15 | 0.124 | 44.5 | 79.7 |
| --- | --- | --- | --- | --- | --- | --- |
|  |  | F3 | 0.017 | 0.072 | 83.0 | 103.7 |
|  |  | F4 | 0.055 | 0.098 | 81.9 | 94.5 |
|  |  | F5 | 0.045 | 0.078 | 97.5 | 98.4 |
|  |  | F6 | 0.011 | 0.039 | 91.4 | 104.3 |
|  |  | F7 | 0.009 | 0.095 | 90.7 | 96.9 |
|  |  | F8 | 0.014 | 0.09 | 86.7 | 112.8 |
|  |  | F9 | −0.007 | 0.026 | 100.1 | 107.4 |
|  |  | F10 | −0.015 | 0.064 | 98.1 | 121.3 |
|  |  | F11 | 0.008 | 0.07 | 91.1 | 107.0 |
|  | **8.9** | F2 | 0.01 | 0.018 | 76.6 | 94.2 |
|  |  | F3 | −0.016 | 0.015 | 87.3 | 101.1 |
|  |  | F4 | 0.021 | 0.024 | 93.3 | 99.4 |
|  |  | F5 | −0.004 | 0.026 | 97.0 | 102.3 |
|  |  | F6 | −0.002 | 0.002 | 97.1 | 112.1 |
|  |  | F7 | −0.027 | −0.032 | 102.7 | 113.6 |
|  |  | F8 | −0.003 | 0.046 | 105.1 | 108.5 |
|  |  | F9 | −0.024 | 0.016 | 87.6 | 108.7 |
|  |  | F10 | −0.01 | −0.014 | 101.2 | 100.5 |
|  |  | F11 | 0.017 | 0.011 | 95.7 | 109.5 |
|  | **4.6** | F2 | 0.007 | 0.024 | 92.9 | 108.9 |
|  |  | F3 | −0.016 | 0.016 | 92.9 | 107.9 |
|  |  | F4 | 0.005 | 0.01 | 97.4 | 101.6 |
|  |  | F5 | −0.02 | 0.025 | 96.0 | 110.9 |
|  |  | F6 | −0.015 | 0.033 | 95.7 | 114.9 |
|  |  | F7 | 0.065 | 0.036 | 93.4 | 118.8 |
|  |  | F8 | 0.031 | 0.051 | 90.5 | 110.8 |
|  |  | F9 | 0.076 | 0.051 | 96.4 | 92.8 |
|  |  | F10 | 0.067 | 0.063 | 94.0 | 109.7 |
|  |  | F11 | 0.069 | 0.065 | 90.4 | 106.1 |
|  | **2.2** | F2 | −0.01 | 0.056 | 93.9 | 105.8 |
|  |  | F3 | 0.032 | 0.016 | 94.4 | 117.7 |
|  |  | F4 | −0.01 | 0.036 | 98.0 | 101.5 |
|  |  | F5 | −0.009 | 0.05 | 96.8 | 115.1 |
|  |  | F6 | 0 | 0.048 | 94.6 | 108.0 |
|  |  | F7 | 0.015 | 0.019 | 89.6 | 121.6 |
|  |  | F8 | 0.009 | −0.002 | 91.1 | 116.0 |
|  |  | F9 | −0.017 | 0.043 | 98.0 | 114.4 |
|  |  | F10 | 0.005 | 0.031 | 102.0 | 119.8 |
|  |  | F11 | −0.001 | 0.026 | 97.7 | 115.9 |

**Table S1.** *Cont.*

| **Elderberry** | **17.8** | F2 | 0.002 | 0.017 | 100.7 | 104.7 |
| --- | --- | --- | --- | --- | --- | --- |
|  |  | F3 | −0.014 | −0.004 | 95.3 | 116.7 |
|  |  | F4 | −0.014 | 0.016 | 92.2 | 107.5 |
|  |  | F5 | 0.009 | 0.017 | 91.2 | 99.0 |
|  |  | F6 | 0.021 | 0.021 | 96.0 | 95.1 |
|  |  | F7 | 0.014 | −0.013 | 104.9 | 95.5 |
|  |  | F8 | 0.106 | 0.119 | 96.3 | 84.5 |
|  |  | F9 | 0.028 | 0.018 | 108.3 | 96.4 |
|  |  | F10 | 0.028 | −0.004 | 108.0 | 109.5 |
|  |  | F11 | 0.03 | 0.006 | 110.6 | 102.4 |
|  | **8.9** | F2 | 0.006 | −0.026 | 101.4 | 107.2 |
|  |  | F3 | 0.004 | −0.024 | 93.5 | 99.4 |
|  |  | F4 | −0.008 | −0.023 | 100.0 | 94.2 |
|  |  | F5 | 0.008 | −0.012 | 94.7 | 83.2 |
|  |  | F6 | 0.008 | 0 | 102.6 | 84.1 |
|  |  | F7 | −0.021 | 0.023 | 89.9 | 96.6 |
|  |  | F8 | 0.049 | 0.057 | 94.7 | 87.9 |
|  |  | F9 | −0.013 | 0.005 | 106.2 | 106.4 |
|  |  | F10 | −0.009 | 0.022 | 96.7 | 103.3 |
|  |  | F11 | −0.023 | 0.019 | 96.7 | 96.6 |
|  | **4.6** | F2 | 0.02 | 0.124 | 97.7 | 111.1 |
|  |  | F3 | 0.023 | 0.108 | 96.0 | 81.0 |
|  |  | F4 | 0.004 | 0.111 | 99.9 | 94.1 |
|  |  | F5 | 0.009 | 0.122 | 101.4 | 85.3 |
|  |  | F6 | 0.012 | 0.116 | 88.5 | 105.5 |
|  |  | F7 | −0.002 | 0.007 | 98.1 | 103.6 |
|  |  | F8 | 0 | 0.002 | 91.5 | 100.1 |
|  |  | F9 | 0.004 | 0.002 | 95.4 | 103.7 |
|  |  | F10 | 0.014 | 0.014 | 96.3 | 88.1 |
|  |  | F11 | 0.011 | 0.001 | 99.9 | 97.1 |
|  | **2.2** | F2 | 0.005 | 0.001 | 90.7 | 94.1 |
|  |  | F3 | −0.005 | −0.009 | 100.7 | 111.7 |
|  |  | F4 | −0.022 | 0.012 | 107.0 | 93.4 |
|  |  | F5 | −0.005 | 0.001 | 97.7 | 94.4 |
|  |  | F6 | 0.007 | 0.002 | 96.1 | 92.3 |
|  |  | F7 | −0.001 | 0.024 | 94.4 | 105.4 |
|  |  | F8 | −0.007 | −0.015 | 95.0 | 101.5 |
|  |  | F9 | −0.006 | −0.015 | 100.0 | 99.8 |
|  |  | F10 | 0 | −0.007 | 106.0 | 100.7 |
|  |  | F11 | −0.003 | −0.012 | 98.3 | 87.5 |

**Table S1.** *Cont.*

| **Strawberry** | **17.8** | F2 | 0.049 | 0.069 | 83.8 | 97.4 |
| --- | --- | --- | --- | --- | --- | --- |
|  |  | F3 | −0.054 | 0.028 | 99.7 | 107.9 |
|  |  | F4 | 0.019 | 0.091 | 93.7 | 100.0 |
|  |  | F5 | 0.041 | 0.086 | 77.9 | 96.1 |
|  |  | F6 | 0.012 | 0.07 | 92.5 | 96.6 |
|  |  | F7 | 0.058 | −0.002 | 93.1 | 107.0 |
|  |  | F8 | 0.037 | −0.024 | 93.4 | 95.5 |
|  |  | F9 | 0.048 | −0.019 | 92.5 | 99.6 |
|  |  | F10 | 0.073 | −0.025 | 107.7 | 98.4 |
|  |  | F11 | 0.09 | −0.005 | 90.2 | 99.3 |
|  | **8.9** | F2 | 0.069 | 0.006 | 82.9 | 99.9 |
|  |  | F3 | −0.021 | 0.051 | 101.0 | 103.4 |
|  |  | F4 | −0.024 | 0.044 | 94.9 | 97.2 |
|  |  | F5 | −0.004 | 0.068 | 83.5 | 79.1 |
|  |  | F6 | −0.012 | 0.049 | 93.7 | 81.4 |
|  |  | F7 | 0.057 | 0.061 | 88.8 | 94.0 |
|  |  | F8 | 0.02 | 0.015 | 92.7 | 100.1 |
|  |  | F9 | 0.032 | 0.059 | 99.7 | 106.8 |
|  |  | F10 | 0.043 | 0.02 | 96.0 | 114.8 |
|  |  | F11 | 0.072 | 0.017 | 87.7 | 87.9 |
|  | **4.6** | F2 | −0.039 | 0.041 | 89.2 | 110.4 |
|  |  | F3 | 0.036 | 0.06 | 93.1 | 106.3 |
|  |  | F4 | 0.026 | 0.082 | 92.6 | 89.5 |
|  |  | F5 | 0.034 | 0.06 | 110.6 | 91.7 |
|  |  | F6 | 0.041 | 0.081 | 78.4 | 99.8 |
|  |  | F7 | 0.014 | −0.024 | 96.7 | 105.3 |
|  |  | F8 | 0 | −0.013 | 100.1 | 109.4 |
|  |  | F9 | −0.011 | 0 | 100.9 | 97.8 |
|  |  | F10 | 0.006 | 0.016 | 108.0 | 91.1 |
|  |  | F11 | 0.021 | 0.012 | 87.2 | 91.4 |
|  | **2.2** | F2 | −0.003 | −0.027 | 98.3 | 98.4 |
|  |  | F3 | 0.003 | −0.059 | 94.2 | 105.5 |
|  |  | F4 | 0.013 | −0.034 | 104.3 | 85.6 |
|  |  | F5 | 0.007 | 0.014 | 100.4 | 101.0 |
|  |  | F6 | 0.012 | −0.022 | 84.3 | 103.2 |
|  |  | F7 | 0.003 | 0.072 | 105.7 | 104.1 |
|  |  | F8 | 0.013 | 0.041 | 104.8 | 110.1 |
|  |  | F9 | 0 | 0.026 | 100.3 | 109.5 |
|  |  | F10 | −0.015 | 0.065 | 114.2 | 116.5 |
|  |  | F11 | 0.017 | 0.072 | 86.5 | 106.4 |

**Table S1.** *Cont.*

| **Cranberry** | **17.8** | F2 | 0.019 | 0.027 | 95.0 | 79.9 |
| --- | --- | --- | --- | --- | --- | --- |
|  |  | F3 | 0.027 | 0.014 | 98.8 | 96.5 |
|  |  | F4 | 0.017 | 0.036 | 100.5 | 98.2 |
|  |  | F5 | 0.025 | 0.022 | 112.6 | 97.2 |
|  |  | F6 | 0.031 | 0.017 | 103.6 | 96.5 |
|  |  | F7 | 0.008 | 0.016 | 101.8 | 89.5 |
|  |  | F8 | 0.154 | 0.136 | 34.7 | 31.7 |
|  |  | F9 | 0.145 | 0.139 | 57.7 | 51.6 |
|  |  | F10 | 0.01 | 0.012 | 106.6 | 95.5 |
|  |  | F11 | 0.01 | 0.023 | 103.9 | 107.1 |
|  | **8.9** | F2 | 0 | −0.023 | 107.5 | 103.3 |
|  |  | F3 | −0.007 | −0.012 | 107.5 | 82.6 |
|  |  | F4 | 0.007 | −0.015 | 96.0 | 84.2 |
|  |  | F5 | 0 | −0.02 | 103.8 | 98.5 |
|  |  | F6 | 0.011 | −0.004 | 95.0 | 88.6 |
|  |  | F7 | 0.003 | 0.007 | 100.2 | 98.1 |
|  |  | F8 | 0.156 | 0.123 | 46.6 | 48.4 |
|  |  | F9 | 0.113 | 0.099 | 93.9 | 88.3 |
|  |  | F10 | 0.033 | 0.012 | 94.2 | 93.4 |
|  |  | F11 | 0.023 | 0.011 | 106.4 | 99.7 |
|  | **4.6** | F2 | −0.008 | −0.011 | 109.3 | 105.3 |
|  |  | F3 | −0.03 | 0.006 | 114.4 | 91.9 |
|  |  | F4 | −0.02 | −0.011 | 100.3 | 91.9 |
|  |  | F5 | −0.015 | −0.002 | 105.1 | 90.7 |
|  |  | F6 | −0.006 | 0.006 | 120.4 | 102.5 |
|  |  | F7 | −0.017 | −0.018 | 97.3 | 91.0 |
|  |  | F8 | 0.083 | 0.074 | 88.4 | 81.8 |
|  |  | F9 | 0 | −0.006 | 92.9 | 97.1 |
|  |  | F10 | 0.007 | −0.04 | 125.4 | 109.3 |
|  |  | F11 | −0.015 | −0.012 | 99.7 | 94.9 |
|  | **2.2** | F2 | −0.001 | −0.008 | 100.0 | 99.7 |
|  |  | F3 | −0.006 | −0.008 | 94.2 | 98.0 |
|  |  | F4 | 0.012 | −0.005 | 106.5 | 103.0 |
|  |  | F5 | 0.012 | −0.007 | 100.2 | 89.6 |
|  |  | F6 | 0.019 | 0.004 | 93.9 | 89.2 |
|  |  | F7 | −0.019 | 0.011 | 97.7 | 93.2 |
|  |  | F8 | 0.014 | 0.008 | 100.2 | 96.0 |
|  |  | F9 | −0.02 | −0.006 | 103.1 | 94.4 |
|  |  | F10 | −0.012 | 0.005 | 107.4 | 93.6 |
|  |  | F11 | −0.015 | 0.03 | 106.4 | 88.9 |

**Table S1.** *Cont.*

| **Red Raspberry** | **17.8** | F2 | 0.117 | 0.237 | 100.8 | 52.1 |
| --- | --- | --- | --- | --- | --- | --- |
|  |  | F3 | −0.002 | 0.067 | 57.6 | 75.9 |
|  |  | F4 | 0.003 | 0.102 | 88.4 | 83.1 |
|  |  | F5 | 0.009 | 0.105 | 97.9 | 76.8 |
|  |  | F6 | −0.011 | 0.079 | 97.6 | 82.5 |
|  |  | F7 | 0.019 | 0.04 | 106.6 | 101.3 |
|  |  | F8 | 0.006 | −0.016 | 105.4 | 104.7 |
|  |  | F9 | −0.005 | −0.023 | 98.2 | 118.9 |
|  |  | F10 | 0.007 | −0.047 | 104.5 | 103.2 |
|  |  | F11 | −0.008 | −0.006 | 96.9 | 83.0 |
|  | **8.9** | F2 | −0.015 | 0.026 | 96.1 | 75.1 |
|  |  | F3 | −0.008 | 0.022 | 103.3 | 90.1 |
|  |  | F4 | −0.012 | 0.025 | 79.2 | 86.7 |
|  |  | F5 | −0.004 | 0.02 | 93.5 | 99.7 |
|  |  | F6 | −0.021 | 0.002 | 95.3 | 84.2 |
|  |  | F7 | −0.011 | 0.051 | 86.2 | 108.4 |
|  |  | F8 | −0.019 | 0.019 | 95.4 | 105.3 |
|  |  | F9 | −0.041 | 0.038 | 85.3 | 99.9 |
|  |  | F10 | −0.047 | 0 | 94.7 | 111.7 |
|  |  | F11 | −0.021 | 0.058 | 100.8 | 96.8 |
|  | **4.6** | F2 | 0.003 | 0.025 | 101.1 | 97.9 |
|  |  | F3 | −0.018 | 0.039 | 90.1 | 94.0 |
|  |  | F4 | −0.022 | 0.018 | 88.9 | 99.9 |
|  |  | F5 | −0.013 | 0.005 | 93.0 | 103.1 |
|  |  | F6 | −0.01 | 0.045 | 101.3 | 91.0 |
|  |  | F7 | 0.046 | −0.021 | 98.8 | 96.4 |
|  |  | F8 | 0.005 | 0.007 | 113.5 | 106.6 |
|  |  | F9 | 0.003 | 0.041 | 91.4 | 94.7 |
|  |  | F10 | 0.001 | 0.004 | 95.2 | 104.3 |
|  |  | F11 | 0.027 | −0.006 | 93.1 | 96.8 |
|  | **2.2** | F2 | 0.031 | 0.037 | 104.6 | 96.2 |
|  |  | F3 | −0.005 | 0.042 | 94.9 | 121.2 |
|  |  | F4 | −0.019 | 0.076 | 90.6 | 88.7 |
|  |  | F5 | 0.009 | 0.078 | 95.5 | 116.5 |
|  |  | F6 | 0 | 0.065 | 103.9 | 107.6 |
|  |  | F7 | −0.043 | 0.005 | 101.5 | 95.9 |
|  |  | F8 | −0.04 | −0.006 | 92.6 | 99.5 |
|  |  | F9 | −0.039 | −0.005 | 86.9 | 98.2 |
|  |  | F10 | −0.026 | −0.016 | 87.0 | 86.1 |
|  |  | F11 | −0.049 | 0.022 | 92.4 | 87.7 |

**Table S1.** *Cont.*

| **Blackcurrant** | **17.8** | F2 | 0.018 | −0.008 | 90.9 | 102.7 |
| --- | --- | --- | --- | --- | --- | --- |
|  |  | F3 | 0.054 | 0.022 | 103.5 | 100.5 |
|  |  | F4 | 0.043 | 0.025 | 100.0 | 97.3 |
|  |  | F5 | 0.007 | −0.012 | 102.8 | 96.3 |
|  |  | F6 | −0.028 | −0.029 | 104.4 | 89.3 |
|  |  | F7 | 0.024 | −0.008 | 103.3 | 98.6 |
|  |  | F8 | −0.034 | −0.014 | 102.2 | 90.7 |
|  |  | F9 | −0.026 | 0.007 | 93.2 | 94.2 |
|  |  | F10 | −0.009 | −0.002 | 97.3 | 87.3 |
|  |  | F11 | −0.021 | 0.021 | 100.2 | 85.2 |
|  | **8.9** | F2 | 0.019 | 0.016 | 95.7 | 104.7 |
|  |  | F3 | 0.028 | 0.019 | 95.6 | 101.0 |
|  |  | F4 | 0 | 0.017 | 101.6 | 105.1 |
|  |  | F5 | 0 | 0.006 | 80.1 | 111.9 |
|  |  | F6 | −0.012 | 0.003 | 93.6 | 98.2 |
|  |  | F7 | 0.007 | 0.021 | 93.7 | 106.7 |
|  |  | F8 | −0.005 | 0.025 | 76.4 | 97.4 |
|  |  | F9 | −0.024 | 0.018 | 84.0 | 103.5 |
|  |  | F10 | −0.016 | 0.023 | 102.6 | 89.2 |
|  |  | F11 | −0.013 | 0.03 | 86.1 | 83.7 |
|  | **4.6** | F2 | −0.01 | 0.021 | 100.4 | 91.1 |
|  |  | F3 | 0.011 | 0.026 | 85.8 | 91.0 |
|  |  | F4 | 0.014 | 0.019 | 91.4 | 99.7 |
|  |  | F5 | 0.022 | 0.034 | 97.9 | 85.1 |
|  |  | F6 | 0.012 | 0.02 | 108.0 | 93.2 |
|  |  | F7 | 0.023 | 0.008 | 111.5 | 92.4 |
|  |  | F8 | −0.032 | 0.024 | 95.3 | 102.0 |
|  |  | F9 | −0.015 | 0.007 | 111.7 | 98.8 |
|  |  | F10 | −0.042 | 0.001 | 108.9 | 90.5 |
|  |  | F11 | −0.022 | 0.017 | 105.0 | 98.0 |
|  | **2.2** | F2 | −0.006 | 0.003 | 106.3 | 109.2 |
|  |  | F3 | −0.011 | −0.002 | 102.2 | 103.5 |
|  |  | F4 | −0.01 | −0.01 | 85.3 | 88.4 |
|  |  | F5 | −0.014 | 0.017 | 88.9 | 90.9 |
|  |  | F6 | −0.016 | 0.003 | 106.4 | 90.6 |
|  |  | F7 | 0.014 | 0.015 | 112.7 | 108.8 |
|  |  | F8 | −0.001 | 0.012 | 93.0 | 106.3 |
|  |  | F9 | −0.006 | 0.01 | 99.9 | 114.2 |
|  |  | F10 | −0.019 | 0.02 | 108.8 | 92.7 |
|  |  | F11 | −0.023 | 0.018 | 107.9 | 116.4 |

**Table S1.** *Cont.*

| **Green Grapes** | **17.8** | F2 | 0.017 | 0.027 | 88.6 | 65.2 |
| --- | --- | --- | --- | --- | --- | --- |
|  |  | F3 | 0.004 | 0.03 | 101.0 | 95.0 |
|  |  | F4 | 0.001 | 0.033 | 94.3 | 75.3 |
|  |  | F5 | 0.008 | 0.006 | 89.6 | 102.0 |
|  |  | F6 | −0.011 | 0.011 | 97.0 | 97.0 |
|  |  | F7 | −0.008 | 0.002 | 88.1 | 96.2 |
|  |  | F8 | −0.006 | −0.004 | 83.6 | 94.1 |
|  |  | F9 | 0.027 | 0.015 | 92.4 | 92.6 |
|  |  | F10 | −0.008 | 0.006 | 88.4 | 108.3 |
|  |  | F11 | 0.006 | 0.003 | 78.4 | 90.9 |
|  | **8.9** | F2 | 0.017 | −0.006 | 81.8 | 101.4 |
|  |  | F3 | 0.001 | −0.014 | 89.9 | 93.6 |
|  |  | F4 | −0.002 | −0.012 | 89.5 | 90.9 |
|  |  | F5 | 0.007 | 0.006 | 88.4 | 90.6 |
|  |  | F6 | 0.002 | −0.018 | 97.0 | 100.0 |
|  |  | F7 | 0.002 | 0.011 | 97.5 | 100.8 |
|  |  | F8 | 0.001 | −0.007 | 96.4 | 91.5 |
|  |  | F9 | 0.017 | 0.017 | 101.7 | 89.1 |
|  |  | F10 | 0.002 | 0 | 112.8 | 120.6 |
|  |  | F11 | 0.002 | 0 | 113.6 | 87.6 |
|  | **4.6** | F2 | −0.003 | 0.029 | 107.2 | 103.3 |
|  |  | F3 | 0.009 | 0.021 | 96.4 | 101.5 |
|  |  | F4 | 0.016 | 0.036 | 94.3 | 93.8 |
|  |  | F5 | 0.013 | 0.017 | 92.8 | 89.5 |
|  |  | F6 | −0.004 | 0.024 | 105.8 | 98.5 |
|  |  | F7 | 0.03 | −0.015 | 95.1 | 91.2 |
|  |  | F8 | 0.02 | −0.005 | 99.5 | 101.1 |
|  |  | F9 | −0.005 | 0 | 92.4 | 87.7 |
|  |  | F10 | 0.017 | −0.022 | 91.1 | 95.3 |
|  |  | F11 | −0.009 | −0.013 | 92.2 | 98.2 |
|  | **2.2** | F2 | 0.006 | 0.005 | 117.5 | 84.2 |
|  |  | F3 | −0.004 | 0.009 | 93.6 | 103.6 |
|  |  | F4 | 0.012 | 0.011 | 93.0 | 92.4 |
|  |  | F5 | 0.001 | −0.002 | 104.8 | 92.1 |
|  |  | F6 | −0.006 | 0.01 | 102.6 | 92.7 |
|  |  | F7 | −0.002 | 0.018 | 87.5 | 99.1 |
|  |  | F8 | −0.015 | −0.003 | 98.4 | 100.5 |
|  |  | F9 | −0.006 | 0.008 | 91.7 | 97.7 |
|  |  | F10 | 0.001 | −0.003 | 90.7 | 123.6 |
|  |  | F11 | −0.035 | 0.021 | 84.9 | 94.4 |

**Table S1.** *Cont.*

| **Black Grapes** | **17.8** | F2 | 0.005 | 0.03 | 69.1 | 80.7 |
| --- | --- | --- | --- | --- | --- | --- |
|  |  | F3 | −0.011 | 0.01 | 85.0 | 83.2 |
|  |  | F4 | −0.009 | 0 | 90.5 | 86.8 |
|  |  | F5 | 0.018 | 0.007 | 86.9 | 86.5 |
|  |  | F6 | −0.003 | 0.005 | 90.2 | 84.9 |
|  |  | F7 | 0.004 | 0.003 | 120.0 | 100.1 |
|  |  | F8 | 0.002 | 0.016 | 98.3 | 111.7 |
|  |  | F9 | 0.014 | 0.06 | 93.7 | 113.1 |
|  |  | F10 | −0.005 | 0.017 | 101.3 | 104.8 |
|  |  | F11 | −0.006 | 0.019 | 101.5 | 97.2 |
|  | **8.9** | F2 | −0.015 | 0.003 | 76.2 | 100.2 |
|  |  | F3 | −0.019 | −0.006 | 89.6 | 93.1 |
|  |  | F4 | −0.013 | 0.005 | 90.5 | 107.3 |
|  |  | F5 | 0.014 | 0.023 | 88.0 | 85.0 |
|  |  | F6 | 0.028 | 0.013 | 93.6 | 97.4 |
|  |  | F7 | −0.001 | 0.005 | 112.7 | 106.5 |
|  |  | F8 | 0.034 | 0.009 | 119.7 | 100.7 |
|  |  | F9 | 0.043 | 0.027 | 97.0 | 92.2 |
|  |  | F10 | 0.036 | 0.003 | 101.8 | 107.2 |
|  |  | F11 | 0.037 | 0.004 | 105.9 | 84.4 |
|  | **4.6** | F2 | 0.012 | 0.021 | 98.1 | 104.3 |
|  |  | F3 | −0.019 | 0.013 | 102.2 | 93.5 |
|  |  | F4 | 0.005 | 0.012 | 93.6 | 107.4 |
|  |  | F5 | 0.012 | −0.009 | 102.8 | 99.9 |
|  |  | F6 | 0.006 | 0.004 | 97.7 | 101.1 |
|  |  | F7 | 0.035 | −0.003 | 101.3 | 99.5 |
|  |  | F8 | 0.011 | 0.013 | 105.1 | 97.0 |
|  |  | F9 | 0.025 | 0.021 | 99.6 | 95.8 |
|  |  | F10 | 0.028 | 0.015 | 94.9 | 96.6 |
|  |  | F11 | 0.056 | 0.014 | 106.7 | 86.5 |
|  | **2.2** | F2 | 0.015 | 0.01 | 99.3 | 100.0 |
|  |  | F3 | −0.009 | 0.02 | 104.0 | 109.2 |
|  |  | F4 | −0.002 | −0.001 | 93.7 | 99.8 |
|  |  | F5 | 0.016 | 0.043 | 92.4 | 102.0 |
|  |  | F6 | 0.004 | 0.035 | 101.9 | 94.4 |
|  |  | F7 | −0.009 | −0.004 | 103.3 | 96.3 |
|  |  | F8 | −0.007 | −0.007 | 106.3 | 90.0 |
|  |  | F9 | −0.003 | −0.004 | 103.8 | 99.5 |
|  |  | F10 | 0 | 0.004 | 104.4 | 110.8 |
|  |  | F11 | 0.009 | 0.012 | 99.3 | 78.0 |

**Table S1.** *Cont.*

| **Pear** | **17.8** | F2 | 0.039 | 0.003 | 0.377 | 67.4 |
| --- | --- | --- | --- | --- | --- | --- |
|  |  | F3 | 0.031 | 0.004 | 0.385 | 94.4 |
|  |  | F4 | 0.055 | 0.009 | 0.361 | 95.0 |
|  |  | F5 | 0.049 | 0.021 | 0.367 | 89.6 |
|  |  | F6 | 0.026 | −0.035 | 0.39 | 96.9 |
|  |  | F7 | 0.028 | −0.024 | 0.414 | 103.2 |
|  |  | F8 | 0.021 | −0.03 | 0.421 | 93.5 |
|  |  | F9 | 0.032 | −0.025 | 0.41 | 98.0 |
|  |  | F10 | 0.037 | −0.04 | 0.405 | 93.8 |
|  |  | F11 | 0.066 | −0.033 | 0.376 | 97.1 |
|  | **8.9** | F2 | 0.013 | 0.024 | 0.385 | 96.9 |
|  |  | F3 | 0.005 | −0.002 | 0.393 | 99.8 |
|  |  | F4 | 0.02 | 0.025 | 0.378 | 103.8 |
|  |  | F5 | 0.038 | 0.027 | 0.36 | 97.7 |
|  |  | F6 | 0.002 | 0.036 | 0.396 | 86.5 |
|  |  | F7 | 0.018 | 0.019 | 0.381 | 101.4 |
|  |  | F8 | −0.005 | 0.004 | 0.404 | 102.2 |
|  |  | F9 | −0.002 | 0.018 | 0.401 | 96.2 |
|  |  | F10 | −0.012 | 0.037 | 0.411 | 91.1 |
|  |  | F11 | 0.027 | 0.021 | 0.372 | 91.7 |
|  | **4.6** | F2 | 0.028 | −0.004 | 0.379 | 104.4 |
|  |  | F3 | 0 | −0.003 | 0.407 | 105.5 |
|  |  | F4 | 0.027 | 0.008 | 0.38 | 99.8 |
|  |  | F5 | 0.018 | 0.007 | 0.389 | 102.6 |
|  |  | F6 | 0.018 | 0.005 | 0.389 | 130.7 |
|  |  | F7 | 0.029 | 0.024 | 0.368 | 105.5 |
|  |  | F8 | −0.033 | 0.002 | 0.43 | 106.5 |
|  |  | F9 | 0.02 | 0 | 0.377 | 104.9 |
|  |  | F10 | 0.001 | −0.019 | 0.396 | 105.5 |
|  |  | F11 | 0.029 | −0.007 | 0.368 | 96.9 |
|  | **2.2** | F2 | 0.003 | 0.004 | 0.434 | 104.9 |
|  |  | F3 | 0.019 | −0.016 | 0.418 | 92.8 |
|  |  | F4 | 0.042 | −0.015 | 0.395 | 84.7 |
|  |  | F5 | 0.034 | −0.025 | 0.403 | 92.9 |
|  |  | F6 | 0.03 | −0.037 | 0.407 | 97.7 |
|  |  | F7 | 0.028 | 0.005 | 0.419 | 105.9 |
|  |  | F8 | 0.037 | −0.011 | 0.41 | 99.9 |
|  |  | F9 | 0.034 | −0.002 | 0.413 | 102.6 |
|  |  | F10 | 0.062 | −0.039 | 0.385 | 93.2 |
|  |  | F11 | 0.05 | −0.009 | 0.397 | 98.4 |

**Table S1.** *Cont.*

| **Mangosteen** | **17.8** | F2 | −0.02 | −0.005 | 98.7 | 93.0 |
| --- | --- | --- | --- | --- | --- | --- |
|  |  | F3 | −0.018 | −0.018 | 94.1 | 87.6 |
|  |  | F4 | −0.025 | −0.008 | 94.1 | 98.2 |
|  |  | F5 | −0.033 | −0.013 | 100.2 | 94.6 |
|  |  | F6 | −0.018 | 0.008 | 103.0 | 98.3 |
|  |  | F7 | 0.01 | 0.01 | 89.3 | 78.0 |
|  |  | F8 | −0.004 | −0.005 | 110.4 | 82.3 |
|  |  | F9 | 0.002 | 0.008 | 112.7 | 92.5 |
|  |  | F10 | 0.056 | 0.06 | 109.1 | 94.3 |
|  |  | F11 | −0.007 | 0.009 | 107.1 | 97.1 |
|  | **8.9** | F2 | −0.018 | −0.014 | 84.9 | 86.5 |
|  |  | F3 | −0.019 | −0.022 | 91.6 | 88.2 |
|  |  | F4 | −0.025 | −0.023 | 113.0 | 91.2 |
|  |  | F5 | −0.013 | −0.013 | 104.5 | 99.0 |
|  |  | F6 | −0.035 | −0.012 | 100.8 | 100.3 |
|  |  | F7 | −0.009 | 0.003 | 87.6 | 80.5 |
|  |  | F8 | −0.003 | −0.004 | 94.2 | 93.5 |
|  |  | F9 | −0.012 | −0.008 | 100.7 | 95.8 |
|  |  | F10 | 0.019 | 0.023 | 90.9 | 102.5 |
|  |  | F11 | 0.003 | −0.001 | 96.1 | 100.8 |
|  | **4.6** | F2 | 0.022 | −0.005 | 86.5 | 84.1 |
|  |  | F3 | 0.023 | −0.029 | 92.1 | 93.5 |
|  |  | F4 | 0.012 | −0.018 | 111.8 | 94.6 |
|  |  | F5 | 0.007 | −0.005 | 107.8 | 97.6 |
|  |  | F6 | 0.005 | −0.004 | 101.5 | 98.3 |
|  |  | F7 | 0.006 | −0.01 | 102.4 | 83.8 |
|  |  | F8 | −0.013 | −0.02 | 90.4 | 93.5 |
|  |  | F9 | −0.001 | −0.02 | 102.8 | 91.6 |
|  |  | F10 | −0.006 | −0.001 | 101.3 | 101.7 |
|  |  | F11 | −0.009 | −0.003 | 99.6 | 97.8 |
|  | **2.2** | F2 | −0.003 | −0.019 | 83.3 | 87.2 |
|  |  | F3 | 0.006 | −0.008 | 101.9 | 93.0 |
|  |  | F4 | 0.016 | −0.018 | 103.8 | 98.9 |
|  |  | F5 | 0.009 | −0.027 | 98.2 | 100.3 |
|  |  | F6 | −0.006 | −0.022 | 102.8 | 101.0 |
|  |  | F7 | 0 | 0 | 90.1 | 90.4 |
|  |  | F8 | −0.023 | 0.003 | 93.2 | 92.5 |
|  |  | F9 | −0.004 | 0.014 | 93.5 | 94.0 |
|  |  | F10 | 0.003 | −0.007 | 95.6 | 98.9 |
|  |  | F11 | −0.007 | 0.004 | 99.2 | 97.8 |

**Table S1.** *Cont.*

| **Plum** | **17.8** | F2 | 0.013 | −0.003 | 105.6 | 99.0 |
| --- | --- | --- | --- | --- | --- | --- |
|  |  | F3 | 0.005 | −0.014 | 97.8 | 99.6 |
|  |  | F4 | 0 | 0.046 | 87.0 | 94.7 |
|  |  | F5 | 0.018 | 0.007 | 87.0 | 96.5 |
|  |  | F6 | −0.016 | 0.052 | 93.5 | 97.9 |
|  |  | F7 | −0.006 | 0.026 | 97.5 | 96.9 |
|  |  | F8 | 0.025 | −0.001 | 89.6 | 101.3 |
|  |  | F9 | 0.02 | 0.026 | 87.1 | 97.5 |
|  |  | F10 | 0.009 | 0.034 | 87.3 | 108.0 |
|  |  | F11 | 0.035 | 0.001 | 93.7 | 82.3 |
|  | **8.9** | F2 | 0.021 | −0.014 | 96.7 | 98.5 |
|  |  | F3 | 0.006 | −0.02 | 100.3 | 96.5 |
|  |  | F4 | 0.043 | −0.012 | 84.6 | 94.2 |
|  |  | F5 | 0.042 | −0.017 | 86.8 | 92.5 |
|  |  | F6 | 0.039 | −0.013 | 83.3 | 92.1 |
|  |  | F7 | −0.031 | −0.021 | 99.7 | 94.5 |
|  |  | F8 | −0.039 | −0.034 | 100.7 | 96.9 |
|  |  | F9 | −0.009 | −0.001 | 99.6 | 94.7 |
|  |  | F10 | −0.025 | −0.014 | 92.4 | 94.8 |
|  |  | F11 | 0.006 | −0.033 | 90.2 | 98.1 |
|  | **4.6** | F2 | −0.009 | −0.002 | 100.8 | 99.3 |
|  |  | F3 | 0.017 | −0.036 | 96.5 | 93.2 |
|  |  | F4 | 0.006 | −0.009 | 92.9 | 93.5 |
|  |  | F5 | −0.018 | −0.007 | 90.0 | 92.4 |
|  |  | F6 | 0.015 | −0.019 | 86.9 | 99.7 |
|  |  | F7 | 0.005 | 0.047 | 92.5 | 95.5 |
|  |  | F8 | 0.002 | 0.003 | 99.6 | 98.6 |
|  |  | F9 | 0.009 | 0.023 | 92.5 | 92.9 |
|  |  | F10 | 0.008 | 0.008 | 87.7 | 95.0 |
|  |  | F11 | −0.015 | 0.004 | 108.8 | 98.0 |
|  | **2.2** | F2 | 0.015 | −0.001 | 102.1 | 93.7 |
|  |  | F3 | −0.011 | −0.018 | 88.3 | 98.5 |
|  |  | F4 | −0.001 | −0.023 | 92.1 | 99.6 |
|  |  | F5 | 0.055 | −0.018 | 88.3 | 96.9 |
|  |  | F6 | 0.046 | −0.04 | 88.3 | 104.1 |
|  |  | F7 | −0.002 | 0.006 | 95.2 | 92.6 |
|  |  | F8 | −0.032 | 0.031 | 103.3 | 96.2 |
|  |  | F9 | 0.005 | 0.017 | 97.6 | 102.1 |
|  |  | F10 | −0.041 | 0.002 | 94.1 | 97.5 |
|  |  | F11 | −0.005 | 0.014 | 84.2 | 93.5 |

**Table S2.** HRK-blue hTLR2 Pam3CysSerLys4 (PAM3CSK4) screening scores for 12 fruits. Quanti-blue (QB) score represents [Solvent control QB score] − [Fraction QB score]. The monosodium salt (WST-1) score represents [WST-1 score for each sample/WST-1 score for the media control (no ligand) *] × 100 which is a percentage.

| **Fruit** | **Concentration (mg/mL)** | **Fraction** | **QB (Replicate 1)** | **QB (Replicate 2)** | **WST-1 (%) (Replicate1)** | **WST-1 (%) (Replicate 2)** |
| --- | --- | --- | --- | --- | --- | --- |
| **Feijoa** | **17.8** | F2 | 0.142 | 0.154 | 97.8 | 103.0 |
|  |  | F3 | 0.199 | 0.234 | 101.0 | 101.6 |
|  |  | F4 | 0.167 | 0.172 | 104.2 | 112.2 |
|  |  | F5 | 0.114 | 0.124 | 104.4 | 111.3 |
|  |  | F6 | 0.023 | −0.015 | 104.2 | 112.7 |
|  |  | F7 | 0.023 | 0.019 | 107.1 | 105.2 |
|  |  | F8 | −0.015 | 0.007 | 109.7 | 110.3 |
|  |  | F9 | 0.042 | 0.043 | 107.7 | 109.7 |
|  |  | F10 | 0.012 | 0.041 | 111.1 | 113.7 |
|  |  | F11 | 0.036 | 0.036 | 106.8 | 111.4 |
|  | **8.9** | F2 | 0.08 | 0.036 | 94.3 | 109.1 |
|  |  | F3 | 0.156 | 0.121 | 96.8 | 112.4 |
|  |  | F4 | 0.12 | 0.091 | 96.9 | 115.6 |
|  |  | F5 | 0.117 | 0.082 | 97.3 | 113.1 |
|  |  | F6 | 0.042 | −0.001 | 89.0 | 119.4 |
|  |  | F7 | 0.01 | −0.017 | 102.2 | 108.7 |
|  |  | F8 | 0.014 | 0.017 | 102.8 | 108.3 |
|  |  | F9 | 0.011 | 0.001 | 104.9 | 112.5 |
|  |  | F10 | 0.001 | 0.023 | 108.9 | 117.8 |
|  |  | F11 | 0.001 | 0.023 | 102.5 | 111.2 |
|  | **4.6** | F2 | 0.051 | 0.028 | 98.0 | 120.0 |
|  |  | F3 | 0.087 | 0.044 | 103.8 | 112.9 |
|  |  | F4 | 0.09 | 0.093 | 103.9 | 115.9 |
|  |  | F5 | 0.046 | 0.04 | 102.9 | 111.2 |
|  |  | F6 | 0.019 | 0.014 | 103.2 | 113.8 |
|  |  | F7 | 0.002 | 0.001 | 100.5 | 101.5 |
|  |  | F8 | −0.015 | −0.016 | 109.1 | 104.8 |
|  |  | F9 | −0.001 | −0.007 | 108.4 | 109.9 |
|  |  | F10 | 0.004 | 0.006 | 107.7 | 119.8 |
|  |  | F11 | −0.007 | 0.031 | 103.8 | 111.4 |
|  | **2.2** | F2 | 0.048 | 0.019 | 104.5 | 105.6 |
|  |  | F3 | 0.041 | 0.041 | 105.0 | 100.8 |
|  |  | F4 | 0.028 | 0.064 | 107.9 | 106.8 |
|  |  | F5 | 0.005 | 0.035 | 106.4 | 114.7 |
|  |  | F6 | −0.019 | 0.039 | 113.9 | 109.1 |
|  |  | F7 | −0.015 | 0.028 | 90.4 | 112.8 |
|  |  | F8 | −0.009 | 0.058 | 101.6 | 109.9 |
|  |  | F9 | −0.023 | 0.064 | 96.8 | 109.9 |
|  |  | F10 | −0.008 | 0.056 | 97.4 | 111.4 |
|  |  | F11 | −0.011 | 0.075 | 96.3 | 101.4 |

**Table S2.** *Cont.*

| **Blackberry** | **17.8** | F2 | 0.008 | −0.001 | 99.5 | 96.5 |
| --- | --- | --- | --- | --- | --- | --- |
|  |  | F3 | 0.021 | −0.048 | 92.3 | 87.7 |
|  |  | F4 | 0.05 | 0.036 | 90.6 | 105.9 |
|  |  | F5 | 0.081 | 0.001 | 90.7 | 109.7 |
|  |  | F6 | 0.03 | 0.004 | 87.8 | 107.0 |
|  |  | F7 | 0.028 | 0.019 | 109.2 | 101.2 |
|  |  | F8 | 0.054 | −0.025 | 106.6 | 95.9 |
|  |  | F9 | 0.055 | 0.035 | 94.8 | 105.3 |
|  |  | F10 | 0.049 | 0.057 | 95.9 | 102.4 |
|  |  | F11 | 0.046 | 0.036 | 99.0 | 109.6 |
|  | **8.9** | F2 | −0.014 | 0.023 | 96.4 | 91.9 |
|  |  | F3 | 0.022 | −0.001 | 93.4 | 98.2 |
|  |  | F4 | 0.044 | 0.086 | 88.5 | 105.9 |
|  |  | F5 | 0.046 | 0.032 | 90.1 | 89.5 |
|  |  | F6 | 0.041 | 0.096 | 87.2 | 104.6 |
|  |  | F7 | 0.011 | 0.009 | 95.7 | 88.9 |
|  |  | F8 | 0.014 | −0.014 | 96.3 | 94.4 |
|  |  | F9 | 0.017 | 0.045 | 95.8 | 98.8 |
|  |  | F10 | 0.058 | 0.017 | 96.4 | 96.8 |
|  |  | F11 | 0.051 | 0.038 | 107.5 | 104.2 |
|  | **4.6** | F2 | −0.023 | 0.002 | 93.0 | 93.8 |
|  |  | F3 | 0.018 | 0.025 | 97.6 | 101.8 |
|  |  | F4 | 0.013 | 0.037 | 100.3 | 105.3 |
|  |  | F5 | 0.024 | 0.02 | 92.3 | 108.3 |
|  |  | F6 | 0.019 | 0.028 | 95.7 | 103.9 |
|  |  | F7 | 0.017 | 0.017 | 93.7 | 88.2 |
|  |  | F8 | 0.012 | 0.018 | 97.7 | 99.1 |
|  |  | F9 | 0.038 | 0.047 | 93.6 | 98.2 |
|  |  | F10 | 0.04 | 0.04 | 92.2 | 108.9 |
|  |  | F11 | 0.055 | 0.044 | 99.9 | 106.1 |
|  | **2.2** | F2 | 0.021 | −0.007 | 89.0 | 96.3 |
|  |  | F3 | 0.008 | 0.008 | 85.0 | 104.5 |
|  |  | F4 | 0.033 | 0.035 | 90.4 | 104.3 |
|  |  | F5 | 0.057 | 0.006 | 86.4 | 104.7 |
|  |  | F6 | 0.052 | 0.02 | 90.5 | 99.0 |
|  |  | F7 | −0.013 | −0.024 | 93.5 | 91.7 |
|  |  | F8 | −0.002 | 0.005 | 94.5 | 96.9 |
|  |  | F9 | 0.004 | −0.049 | 95.4 | 98.0 |
|  |  | F10 | 0.022 | 0.026 | 95.9 | 108.2 |
|  |  | F11 | 0.048 | 0.012 | 98.8 | 97.8 |

**Table S2.** *Cont.*

| **Elderberry** | **17.8** | F2 | −0.009 | −0.022 | 93.4 | 101.7 |
| --- | --- | --- | --- | --- | --- | --- |
|  |  | F3 | 0.018 | −0.009 | 91.2 | 108.9 |
|  |  | F4 | 0.012 | −0.03 | 87.4 | 107.3 |
|  |  | F5 | 0.002 | −0.019 | 87.1 | 97.7 |
|  |  | F6 | 0.002 | −0.011 | 86.2 | 99.6 |
|  |  | F7 | −0.007 | 0 | 86.2 | 97.8 |
|  |  | F8 | 0.009 | 0.009 | 85.4 | 102.3 |
|  |  | F9 | 0.002 | −0.029 | 82.6 | 102.5 |
|  |  | F10 | 0.012 | −0.006 | 92.7 | 95.2 |
|  |  | F11 | 0.001 | 0.001 | 83.6 | 97.8 |
|  | **8.9** | F2 | 0.022 | 0.036 | 88.0 | 100.9 |
|  |  | F3 | −0.002 | 0.041 | 94.2 | 108.7 |
|  |  | F4 | 0.013 | 0.054 | 83.7 | 112.7 |
|  |  | F5 | −0.02 | 0.043 | 83.5 | 108.9 |
|  |  | F6 | −0.007 | 0.041 | 88.0 | 100.1 |
|  |  | F7 | 0 | 0.017 | 98.9 | 97.7 |
|  |  | F8 | 0.013 | 0.024 | 87.7 | 100.4 |
|  |  | F9 | 0.015 | 0.021 | 82.8 | 103.8 |
|  |  | F10 | 0.015 | 0.021 | 86.3 | 110.8 |
|  |  | F11 | 0.015 | 0.036 | 79.3 | 100.8 |
|  | **4.6** | F2 | −0.038 | 0.016 | 92.2 | 95.5 |
|  |  | F3 | −0.009 | 0.025 | 89.2 | 105.6 |
|  |  | F4 | −0.004 | 0.036 | 85.8 | 109.7 |
|  |  | F5 | −0.018 | 0.026 | 87.9 | 103.2 |
|  |  | F6 | −0.044 | 0.036 | 88.2 | 96.6 |
|  |  | F7 | 0.052 | 0.026 | 93.3 | 93.7 |
|  |  | F8 | 0.04 | 0.018 | 87.8 | 102.2 |
|  |  | F9 | 0.063 | 0.029 | 84.4 | 103.4 |
|  |  | F10 | 0.05 | 0.023 | 93.3 | 97.8 |
|  |  | F11 | 0.062 | 0.026 | 84.9 | 96.9 |
|  | **2.2** | F2 | 0.036 | 0.002 | 98.8 | 96.6 |
|  |  | F3 | 0.052 | 0.008 | 90.8 | 103.4 |
|  |  | F4 | 0.018 | −0.008 | 89.5 | 109.2 |
|  |  | F5 | 0.045 | 0.022 | 93.0 | 92.6 |
|  |  | F6 | −0.015 | −0.008 | 85.8 | 94.7 |
|  |  | F7 | 0.012 | 0.006 | 97.7 | 89.8 |
|  |  | F8 | 0.021 | 0.013 | 99.7 | 99.4 |
|  |  | F9 | −0.002 | 0.031 | 102.3 | 105.1 |
|  |  | F10 | 0.025 | 0.029 | 101.5 | 100.8 |
|  |  | F11 | 0.002 | −0.008 | 97.6 | 101.4 |

**Table S2.** *Cont.*

| **Strawberry** | **17.8** | F2 | 0.002 | 0.031 | 93.8 | 90.2 |
| --- | --- | --- | --- | --- | --- | --- |
|  |  | F3 | 0.052 | 0.032 | 97.1 | 95.6 |
|  |  | F4 | 0.075 | 0.104 | 105.8 | 96.5 |
|  |  | F5 | 0.100 | 0.095 | 102.9 | 103.5 |
|  |  | F6 | 0.010 | 0.042 | 111.4 | 97.1 |
|  |  | F7 | 0.010 | 0.031 | 106.8 | 91.3 |
|  |  | F8 | 0.043 | 0.022 | 100.7 | 92.0 |
|  |  | F9 | −0.022 | 0.009 | 111.7 | 90.1 |
|  |  | F10 | −0.009 | −0.002 | 108.7 | 88.1 |
|  |  | F11 | 0.022 | 0.015 | 109.1 | 90.5 |
|  | **8.9** | F2 | 0.003 | 0.023 | 95.8 | 83.9 |
|  |  | F3 | 0.008 | −0.014 | 97.2 | 100.6 |
|  |  | F4 | 0.040 | 0.031 | 99.5 | 101.1 |
|  |  | F5 | 0.059 | 0.082 | 98.8 | 93.1 |
|  |  | F6 | −0.005 | 0.032 | 102.9 | 100.8 |
|  |  | F7 | 0.015 | 0.009 | 98.8 | 88.5 |
|  |  | F8 | 0.017 | −0.004 | 93.2 | 94.0 |
|  |  | F9 | 0.003 | −0.021 | 103.2 | 93.3 |
|  |  | F10 | 0.010 | −0.004 | 104.9 | 95.4 |
|  |  | F11 | 0.006 | −0.005 | 105.4 | 96.7 |
|  | **4.6** | F2 | −0.002 | −0.004 | 93.6 | 89.8 |
|  |  | F3 | 0.041 | 0.010 | 98.0 | 102.0 |
|  |  | F4 | 0.066 | 0.030 | 98.9 | 93.2 |
|  |  | F5 | 0.054 | 0.042 | 101.8 | 94.8 |
|  |  | F6 | 0.019 | −0.012 | 103.7 | 97.6 |
|  |  | F7 | −0.001 | 0.013 | 97.9 | 85.9 |
|  |  | F8 | −0.014 | 0.023 | 106.0 | 91.3 |
|  |  | F9 | −0.025 | −0.020 | 104.3 | 96.9 |
|  |  | F10 | −0.047 | 0.018 | 100.2 | 95.3 |
|  |  | F11 | 0.004 | −0.008 | 98.1 | 91.3 |
|  | **2.2** | F2 | −0.032 | −0.026 | 98.5 | 96.7 |
|  |  | F3 | −0.008 | −0.008 | 99.6 | 98.4 |
|  |  | F4 | −0.004 | −0.024 | 100.4 | 94.1 |
|  |  | F5 | 0.013 | 0.038 | 103.1 | 95.3 |
|  |  | F6 | −0.003 | 0.003 | 107.8 | 96.9 |
|  |  | F7 | 0.018 | 0.040 | 94.5 | 81.6 |
|  |  | F8 | −0.002 | −0.033 | 99.5 | 89.2 |
|  |  | F9 | 0.000 | −0.013 | 99.5 | 90.4 |
|  |  | F10 | 0.022 | −0.030 | 101.9 | 96.7 |
|  |  | F11 | −0.008 | 0.002 | 103.9 | 89.7 |

**Table S2.** *Cont.*

| **Cranberry** | **17.8** | F2 | −0.008 | 0.003 | 117.5 | 96.2 |
| --- | --- | --- | --- | --- | --- | --- |
|  |  | F3 | −0.024 | −0.018 | 109.8 | 100.9 |
|  |  | F4 | −0.015 | −0.006 | 101.7 | 96.2 |
|  |  | F5 | −0.043 | −0.002 | 101.8 | 95.6 |
|  |  | F6 | −0.018 | 0.023 | 105.6 | 88.4 |
|  |  | F7 | 0.007 | 0.02 | 100.1 | 91.7 |
|  |  | F8 | 0.017 | 0.008 | 106.1 | 95.4 |
|  |  | F9 | −0.009 | −0.001 | 119.5 | 99.1 |
|  |  | F10 | 0.026 | −0.047 | 123.8 | 97.6 |
|  |  | F11 | 0.018 | −0.015 | 129.0 | 103.7 |
|  | **8.9** | F2 | 0.011 | 0.022 | 108.4 | 97.9 |
|  |  | F3 | −0.032 | −0.001 | 108.0 | 102.1 |
|  |  | F4 | −0.045 | 0.008 | 108.1 | 91.4 |
|  |  | F5 | −0.029 | −0.008 | 103.2 | 91.9 |
|  |  | F6 | 0.02 | 0.001 | 102.1 | 90.8 |
|  |  | F7 | 0.013 | −0.028 | 124.9 | 95.6 |
|  |  | F8 | 0.038 | 0.003 | 111.8 | 103.4 |
|  |  | F9 | 0.022 | −0.012 | 121.0 | 106.4 |
|  |  | F10 | 0.048 | −0.053 | 112.0 | 102.1 |
|  |  | F11 | −0.003 | 0 | 120.2 | 103.9 |
|  | **4.6** | F2 | −0.03 | −0.005 | 114.9 | 102.8 |
|  |  | F3 | −0.064 | 0.041 | 103.9 | 102.7 |
|  |  | F4 | −0.043 | −0.001 | 104.5 | 89.5 |
|  |  | F5 | −0.058 | −0.02 | 100.8 | 89.1 |
|  |  | F6 | −0.062 | 0.03 | 105.9 | 102.5 |
|  |  | F7 | 0.031 | −0.009 | 119.6 | 91.4 |
|  |  | F8 | 0.044 | −0.031 | 105.3 | 95.9 |
|  |  | F9 | 0.01 | −0.01 | 107.8 | 98.5 |
|  |  | F10 | 0.018 | −0.057 | 108.8 | 101.4 |
|  |  | F11 | 0.009 | −0.033 | 108.8 | 102.9 |
|  | **2.2** | F2 | −0.053 | −0.02 | 118.5 | 95.9 |
|  |  | F3 | −0.023 | −0.008 | 100.9 | 102.4 |
|  |  | F4 | −0.06 | −0.007 | 101.0 | 92.7 |
|  |  | F5 | −0.071 | 0.015 | 106.5 | 92.0 |
|  |  | F6 | −0.033 | 0.014 | 106.9 | 100.2 |
|  |  | F7 | 0.036 | 0.035 | 121.8 | 84.6 |
|  |  | F8 | 0.025 | 0.028 | 111.1 | 89.5 |
|  |  | F9 | 0.009 | −0.023 | 113.5 | 91.4 |
|  |  | F10 | 0.035 | 0 | 115.1 | 98.1 |
|  |  | F11 | 0.029 | −0.015 | 111.7 | 99.2 |

**Table S2.** *Cont.*

| **Red Raspberry** | **17.8** | F2 | 0.009 | 0.033 | 129.3 | 110.8 |
| --- | --- | --- | --- | --- | --- | --- |
|  |  | F3 | −0.007 | −0.002 | 104.4 | 91.7 |
|  |  | F4 | 0.046 | 0.049 | 103.0 | 105.2 |
|  |  | F5 | 0.034 | 0.031 | 117.1 | 107.3 |
|  |  | F6 | −0.024 | 0.029 | 102.2 | 111.6 |
|  |  | F7 | 0.009 | −0.021 | 102.6 | 91.2 |
|  |  | F8 | −0.029 | −0.031 | 103.9 | 106.7 |
|  |  | F9 | −0.016 | 0.002 | 102.9 | 105.5 |
|  |  | F10 | −0.016 | −0.035 | 105.8 | 103.3 |
|  |  | F11 | −0.033 | −0.067 | 117.1 | 110.4 |
|  | **8.9** | F2 | 0.033 | 0.042 | 118.3 | 103.2 |
|  |  | F3 | 0.016 | 0.016 | 115.2 | 95.1 |
|  |  | F4 | 0.048 | 0.032 | 115.0 | 107.6 |
|  |  | F5 | 0.062 | 0.045 | 115.3 | 115.1 |
|  |  | F6 | 0.001 | −0.005 | 112.4 | 113.0 |
|  |  | F7 | 0.006 | −0.024 | 102.4 | 98.1 |
|  |  | F8 | −0.01 | −0.015 | 100.9 | 100.7 |
|  |  | F9 | 0.004 | 0.01 | 102.3 | 114.8 |
|  |  | F10 | −0.035 | −0.033 | 111.3 | 110.0 |
|  |  | F11 | −0.035 | −0.026 | 116.5 | 105.5 |
|  | **4.6** | F2 | 0.013 | 0.007 | 142.7 | 96.8 |
|  |  | F3 | 0.028 | −0.021 | 107.8 | 105.7 |
|  |  | F4 | 0.02 | −0.015 | 102.5 | 107.8 |
|  |  | F5 | 0.026 | −0.004 | 116.6 | 109.6 |
|  |  | F6 | 0.007 | −0.043 | 113.9 | 118.1 |
|  |  | F7 | 0.009 | 0.032 | 101.5 | 95.4 |
|  |  | F8 | −0.017 | 0.024 | 98.3 | 96.8 |
|  |  | F9 | −0.047 | 0.029 | 99.8 | 109.2 |
|  |  | F10 | −0.026 | −0.003 | 105.9 | 110.3 |
|  |  | F11 | −0.024 | 0.008 | 113.6 | 112.5 |
|  | **2.2** | F2 | 0.003 | 0.02 | 107.2 | 99.0 |
|  |  | F3 | 0.017 | 0.028 | 104.1 | 110.9 |
|  |  | F4 | 0.003 | 0.034 | 116.7 | 115.2 |
|  |  | F5 | 0 | 0.034 | 108.2 | 116.0 |
|  |  | F6 | 0.007 | 0.032 | 113.4 | 132.6 |
|  |  | F7 | −0.012 | 0.017 | 98.1 | 92.7 |
|  |  | F8 | 0.011 | 0.024 | 114.6 | 84.9 |
|  |  | F9 | −0.016 | 0.018 | 93.2 | 96.0 |
|  |  | F10 | 0.008 | −0.031 | 101.2 | 103.7 |
|  |  | F11 | 0 | −0.023 | 112.9 | 104.8 |

**Table S2.** *Cont.*

| **Blackcurrant** | **17.8** | F2 | 0.016 | 0.008 | 128.0 | 101.6 |
| --- | --- | --- | --- | --- | --- | --- |
|  |  | F3 | 0.024 | 0.028 | 121.1 | 120.7 |
|  |  | F4 | 0.011 | 0.023 | 95.9 | 105.5 |
|  |  | F5 | 0.006 | 0.022 | 96.4 | 111.7 |
|  |  | F6 | 0.001 | 0.015 | 113.6 | 135.1 |
|  |  | F7 | −0.001 | 0.006 | 137.8 | 113.0 |
|  |  | F8 | 0 | −0.013 | 107.3 | 110.3 |
|  |  | F9 | −0.027 | −0.001 | 104.2 | 140.6 |
|  |  | F10 | 0.017 | −0.001 | 136.2 | 108.0 |
|  |  | F11 | −0.014 | 0.01 | 98.3 | 105.9 |
|  | **8.9** | F2 | 0.007 | 0.009 | 163.6 | 120.5 |
|  |  | F3 | 0.008 | 0.006 | 123.4 | 103.1 |
|  |  | F4 | 0.008 | −0.001 | 115.5 | 117.2 |
|  |  | F5 | −0.004 | 0.003 | 113.4 | 98.9 |
|  |  | F6 | −0.038 | 0.003 | 110.6 | 110.9 |
|  |  | F7 | −0.009 | 0.039 | 112.7 | 109.2 |
|  |  | F8 | −0.007 | 0.023 | 119.8 | 126.8 |
|  |  | F9 | 0.031 | 0.031 | 98.5 | 178.3 |
|  |  | F10 | 0.022 | 0.027 | 95.3 | 116.2 |
|  |  | F11 | 0.032 | 0.036 | 94.4 | 104.8 |
|  | **4.6** | F2 | 0.006 | −0.004 | 114.6 | 136.0 |
|  |  | F3 | 0.021 | 0.019 | 142.0 | 115.7 |
|  |  | F4 | 0.025 | 0.016 | 102.7 | 122.0 |
|  |  | F5 | 0.015 | 0.025 | 103.3 | 143.9 |
|  |  | F6 | 0.002 | 0.022 | 102.3 | 134.1 |
|  |  | F7 | 0.007 | 0.012 | 151.7 | 104.1 |
|  |  | F8 | 0.008 | −0.007 | 122.7 | 106.8 |
|  |  | F9 | 0.015 | −0.017 | 108.6 | 122.4 |
|  |  | F10 | 0.006 | −0.001 | 98.8 | 110.9 |
|  |  | F11 | 0.02 | −0.016 | 98.7 | 105.9 |
|  | **2.2** | F2 | 0.002 | −0.004 | 145.5 | 123.8 |
|  |  | F3 | 0.021 | 0.023 | 173.9 | 127.4 |
|  |  | F4 | 0.015 | 0.005 | 113.4 | 168.0 |
|  |  | F5 | 0.008 | 0.011 | 138.3 | 132.7 |
|  |  | F6 | 0.013 | 0.007 | 117.7 | 128.9 |
|  |  | F7 | 0.011 | 0.027 | 91.7 | 135.1 |
|  |  | F8 | −0.009 | 0.013 | 105.0 | 106.6 |
|  |  | F9 | −0.007 | 0.032 | 92.7 | 121.9 |
|  |  | F10 | −0.006 | 0.011 | 110.4 | 107.9 |
|  |  | F11 | 0.008 | 0.024 | 103.6 | 117.1 |

**Table S2.** *Cont.*

| **Green Grapes** | **17.8** | F2 | 0.054 | 0.032 | 85.0 | 95.3 |
| --- | --- | --- | --- | --- | --- | --- |
|  |  | F3 | 0.048 | 0.013 | 90.1 | 98.3 |
|  |  | F4 | 0.048 | 0.011 | 93.5 | 96.8 |
|  |  | F5 | 0.053 | 0.004 | 84.6 | 97.4 |
|  |  | F6 | 0.04 | 0.011 | 90.1 | 99.5 |
|  |  | F7 | −0.009 | 0.019 | 93.9 | 103.6 |
|  |  | F8 | 0.023 | 0.012 | 95.5 | 107.9 |
|  |  | F9 | 0.009 | −0.02 | 97.4 | 100.6 |
|  |  | F10 | 0.006 | 0.011 | 97.0 | 97.9 |
|  |  | F11 | −0.002 | −0.013 | 92.7 | 90.0 |
|  | **8.9** | F2 | −0.006 | 0.055 | 86.4 | 96.6 |
|  |  | F3 | 0.01 | 0.043 | 84.4 | 93.7 |
|  |  | F4 | 0.021 | 0.038 | 94.1 | 93.3 |
|  |  | F5 | −0.003 | 0.021 | 91.9 | 98.1 |
|  |  | F6 | −0.02 | −0.01 | 95.7 | 100.6 |
|  |  | F7 | 0.025 | 0.012 | 98.8 | 100.8 |
|  |  | F8 | 0.018 | 0.029 | 97.6 | 102.3 |
|  |  | F9 | 0.016 | 0.033 | 104.3 | 101.0 |
|  |  | F10 | 0.006 | 0.046 | 112.4 | 93.9 |
|  |  | F11 | 0.008 | 0.052 | 103.0 | 80.9 |
|  | **4.6** | F2 | 0.015 | 0.014 | 96.1 | 101.7 |
|  |  | F3 | 0.004 | 0.01 | 91.5 | 102.1 |
|  |  | F4 | 0.03 | 0.022 | 93.3 | 101.7 |
|  |  | F5 | 0.009 | −0.009 | 89.9 | 100.6 |
|  |  | F6 | 0.024 | 0.024 | 91.5 | 99.7 |
|  |  | F7 | 0.04 | 0.021 | 99.2 | 101.9 |
|  |  | F8 | 0.025 | 0.03 | 107.7 | 100.8 |
|  |  | F9 | 0.029 | 0.018 | 103.7 | 97.9 |
|  |  | F10 | 0.027 | 0.028 | 105.1 | 91.9 |
|  |  | F11 | 0.01 | 0.022 | 102.6 | 85.8 |
|  | **2.2** | F2 | 0.039 | 0.032 | 92.9 | 100.6 |
|  |  | F3 | 0.032 | 0.049 | 88.2 | 106.7 |
|  |  | F4 | 0.033 | 0.02 | 85.8 | 97.2 |
|  |  | F5 | 0.044 | 0.037 | 89.5 | 93.5 |
|  |  | F6 | 0.051 | 0.032 | 90.9 | 100.5 |
|  |  | F7 | 0.023 | 0.023 | 103.4 | 101.4 |
|  |  | F8 | 0.019 | 0.012 | 105.5 | 104.5 |
|  |  | F9 | 0.022 | 0.026 | 106.1 | 89.9 |
|  |  | F10 | 0.038 | 0.036 | 105.7 | 97.0 |
|  |  | F11 | 0.022 | 0.014 | 107.3 | 91.3 |

**Table S2.** *Cont.*

| **Black Grapes** | **17.8** | F2 | 0.067 | 0.024 | 100.6 | 97.0 |
| --- | --- | --- | --- | --- | --- | --- |
|  |  | F3 | 0.022 | 0.008 | 93.6 | 102.5 |
|  |  | F4 | 0.007 | 0.032 | 104.2 | 111.6 |
|  |  | F5 | 0.015 | 0.024 | 94.8 | 114.2 |
|  |  | F6 | −0.006 | −0.004 | 109.0 | 102.4 |
|  |  | F7 | 0.001 | −0.008 | 96.6 | 107.9 |
|  |  | F8 | 0.022 | 0.016 | 102.2 | 115.4 |
|  |  | F9 | 0.013 | 0.018 | 111.4 | 122.6 |
|  |  | F10 | 0.018 | 0.033 | 114.5 | 114.8 |
|  |  | F11 | −0.026 | 0.025 | 108.3 | 107.2 |
|  | **8.9** | F2 | −0.002 | 0.063 | 108.7 | 102.7 |
|  |  | F3 | 0.005 | 0.018 | 105.2 | 114.1 |
|  |  | F4 | −0.003 | 0.039 | 112.6 | 117.2 |
|  |  | F5 | −0.023 | 0.048 | 112.1 | 127.5 |
|  |  | F6 | −0.039 | −0.012 | 119.7 | 120.0 |
|  |  | F7 | 0.004 | 0.012 | 103.4 | 108.6 |
|  |  | F8 | 0.021 | 0.03 | 111.3 | 112.0 |
|  |  | F9 | 0.003 | 0.034 | 113.5 | 116.2 |
|  |  | F10 | −0.018 | 0.072 | 116.5 | 118.4 |
|  |  | F11 | −0.022 | 0.03 | 107.1 | 122.0 |
|  | **4.6** | F2 | 0.009 | −0.028 | 102.6 | 103.9 |
|  |  | F3 | 0.005 | −0.005 | 100.1 | 109.2 |
|  |  | F4 | −0.005 | −0.05 | 105.7 | 112.8 |
|  |  | F5 | −0.023 | −0.03 | 106.1 | 118.7 |
|  |  | F6 | −0.035 | −0.003 | 113.6 | 111.3 |
|  |  | F7 | −0.006 | 0.008 | 108.8 | 99.3 |
|  |  | F8 | −0.013 | 0.029 | 115.1 | 104.8 |
|  |  | F9 | 0.032 | 0.028 | 113.0 | 110.6 |
|  |  | F10 | 0.037 | 0.027 | 112.9 | 116.8 |
|  |  | F11 | −0.016 | 0.04 | 111.4 | 110.8 |
|  | **2.2** | F2 | 0.024 | −0.014 | 113.1 | 110.5 |
|  |  | F3 | 0.038 | −0.011 | 106.9 | 121.4 |
|  |  | F4 | 0.018 | −0.012 | 106.2 | 117.5 |
|  |  | F5 | −0.027 | −0.049 | 102.6 | 123.9 |
|  |  | F6 | −0.036 | 0.005 | 118.1 | 110.9 |
|  |  | F7 | 0.011 | 0.021 | 113.6 | 109.0 |
|  |  | F8 | 0.015 | 0.013 | 100.4 | 98.2 |
|  |  | F9 | 0.016 | 0.016 | 100.0 | 101.4 |
|  |  | F10 | 0.01 | −0.017 | 108.4 | 106.9 |
|  |  | F11 | 0.017 | 0.029 | 109.0 | 107.0 |

**Table S2.** *Cont.*

| **Pear** | **17.8** | F2 | 0.001 | −0.009 | 118.1 | 111.0 |
| --- | --- | --- | --- | --- | --- | --- |
|  |  | F3 | 0.039 | −0.025 | 95.7 | 105.1 |
|  |  | F4 | 0.014 | −0.005 | 93.2 | 100.2 |
|  |  | F5 | 0.016 | 0.036 | 97.0 | 98.6 |
|  |  | F6 | −0.031 | 0.063 | 96.8 | 104.3 |
|  |  | F7 | 0.005 | 0.025 | 117.4 | 115.7 |
|  |  | F8 | −0.003 | 0.009 | 100.5 | 93.9 |
|  |  | F9 | −0.011 | −0.028 | 101.3 | 89.9 |
|  |  | F10 | −0.001 | 0.01 | 102.7 | 100.3 |
|  |  | F11 | −0.019 | −0.006 | 101.9 | 101.7 |
|  | **8.9** | F2 | 0.032 | 0.042 | 116.3 | 113.8 |
|  |  | F3 | 0.004 | −0.003 | 94.4 | 100.6 |
|  |  | F4 | −0.001 | 0.028 | 89.2 | 94.4 |
|  |  | F5 | 0.006 | 0.023 | 89.4 | 97.0 |
|  |  | F6 | 0.02 | 0.056 | 104.3 | 102.0 |
|  |  | F7 | 0.005 | 0.015 | 93.2 | 90.2 |
|  |  | F8 | −0.022 | 0.04 | 99.0 | 102.4 |
|  |  | F9 | −0.028 | 0.018 | 99.5 | 103.9 |
|  |  | F10 | −0.029 | −0.012 | 107.6 | 101.4 |
|  |  | F11 | −0.068 | 0.026 | 129.3 | 113.2 |
|  | **4.6** | F2 | 0.031 | 0.063 | 110.2 | 111.1 |
|  |  | F3 | −0.009 | 0.059 | 99.0 | 93.8 |
|  |  | F4 | 0.008 | 0.023 | 99.2 | 98.7 |
|  |  | F5 | 0.019 | 0.073 | 92.1 | 102.7 |
|  |  | F6 | −0.015 | 0.021 | 111.5 | 106.1 |
|  |  | F7 | 0.017 | 0.003 | 95.3 | 111.7 |
|  |  | F8 | 0.015 | 0.014 | 96.5 | 91.7 |
|  |  | F9 | 0.007 | −0.006 | 97.1 | 100.4 |
|  |  | F10 | −0.007 | −0.039 | 110.1 | 106.9 |
|  |  | F11 | −0.021 | −0.012 | 128.2 | 108.2 |
|  | **2.2** | F2 | 0.015 | 0.042 | 120.0 | 110.9 |
|  |  | F3 | 0.048 | 0.02 | 89.3 | 109.7 |
|  |  | F4 | 0.007 | 0.005 | 96.1 | 133.5 |
|  |  | F5 | 0.008 | 0.008 | 108.4 | 135.3 |
|  |  | F6 | 0.009 | −0.008 | 114.1 | 141.4 |
|  |  | F7 | 0.009 | −0.004 | 92.3 | 106.7 |
|  |  | F8 | 0.011 | −0.004 | 90.9 | 88.2 |
|  |  | F9 | 0.02 | −0.012 | 89.0 | 103.5 |
|  |  | F10 | 0.029 | 0.006 | 116.2 | 107.5 |
|  |  | F11 | −0.009 | −0.01 | 119.6 | 108.6 |

**Table S2.** *Cont.*

| **Mangosteen** | **17.8** | F2 | −0.013 | 0 | 93.9 | 89.3 |
| --- | --- | --- | --- | --- | --- | --- |
|  |  | F3 | 0.032 | −0.024 | 94.7 | 94.3 |
|  |  | F4 | 0.002 | 0.013 | 89.8 | 91.5 |
|  |  | F5 | −0.024 | −0.008 | 86.6 | 95.0 |
|  |  | F6 | −0.051 | −0.009 | 89.9 | 97.4 |
|  |  | F7 | −0.002 | 0.006 | 103.0 | 110.4 |
|  |  | F8 | 0.001 | −0.023 | 90.2 | 106.3 |
|  |  | F9 | 0.002 | −0.048 | 93.4 | 92.0 |
|  |  | F10 | 0.027 | −0.043 | 94.3 | 96.2 |
|  |  | F11 | 0.001 | −0.025 | 93.9 | 106.4 |
|  | **8.9** | F2 | −0.018 | −0.004 | 98.7 | 99.9 |
|  |  | F3 | −0.024 | −0.027 | 99.9 | 96.4 |
|  |  | F4 | −0.008 | −0.007 | 97.3 | 89.3 |
|  |  | F5 | −0.085 | −0.022 | 98.6 | 100.2 |
|  |  | F6 | −0.063 | −0.042 | 101.3 | 110.2 |
|  |  | F7 | 0.05 | −0.001 | 90.4 | 88.7 |
|  |  | F8 | 0.016 | 0.029 | 98.9 | 96.4 |
|  |  | F9 | 0.054 | 0.058 | 99.1 | 119.4 |
|  |  | F10 | 0.069 | 0.013 | 98.2 | 106.4 |
|  |  | F11 | −0.002 | −0.07 | 101.4 | 99.6 |
|  | **4.6** | F2 | −0.001 | −0.026 | 92.3 | 100.7 |
|  |  | F3 | 0.008 | −0.04 | 104.4 | 100.1 |
|  |  | F4 | −0.062 | −0.016 | 105.0 | 104.6 |
|  |  | F5 | −0.102 | −0.024 | 105.1 | 105.8 |
|  |  | F6 | −0.06 | −0.039 | 102.7 | 105.9 |
|  |  | F7 | 0.065 | −0.015 | 94.4 | 87.1 |
|  |  | F8 | 0.029 | −0.023 | 102.8 | 86.4 |
|  |  | F9 | 0.039 | 0.007 | 103.5 | 101.7 |
|  |  | F10 | 0.032 | −0.065 | 102.0 | 102.9 |
|  |  | F11 | 0.033 | −0.07 | 101.8 | 97.1 |
|  | **2.2** | F2 | −0.077 | −0.041 | 97.1 | 94.4 |
|  |  | F3 | −0.061 | −0.003 | 97.5 | 103.3 |
|  |  | F4 | −0.079 | −0.05 | 96.4 | 96.3 |
|  |  | F5 | −0.053 | −0.025 | 99.4 | 112.3 |
|  |  | F6 | −0.073 | −0.02 | 101.6 | 104.1 |
|  |  | F7 | −0.029 | 0.01 | 90.7 | 86.8 |
|  |  | F8 | −0.01 | 0.002 | 94.0 | 83.9 |
|  |  | F9 | −0.025 | −0.046 | 93.6 | 89.7 |
|  |  | F10 | −0.03 | −0.07 | 92.7 | 92.8 |
|  |  | F11 | −0.056 | −0.027 | 99.3 | 97.5 |

**Table S2.** *Cont.*

| **Plum** | **17.8** | F2 | −0.005 | 0.012 | 106.8 | 90.9 |
| --- | --- | --- | --- | --- | --- | --- |
|  |  | F3 | −0.01 | −0.003 | 101.8 | 92.9 |
|  |  | F4 | 0.005 | 0.017 | 100.9 | 85.4 |
|  |  | F5 | 0.013 | 0.008 | 96.6 | 92.4 |
|  |  | F6 | 0.013 | 0.022 | 98.2 | 97.6 |
|  |  | F7 | 0.013 | 0.042 | 103.2 | 92.2 |
|  |  | F8 | 0.027 | 0.002 | 100.8 | 102.8 |
|  |  | F9 | 0.014 | −0.001 | 111.1 | 104.5 |
|  |  | F10 | 0.038 | 0.014 | 101.6 | 82.7 |
|  |  | F11 | 0.008 | 0.015 | 99.9 | 86.1 |
|  | **8.9** | F2 | 0.008 | 0.014 | 104.5 | 92.0 |
|  |  | F3 | 0.012 | 0.016 | 99.8 | 92.3 |
|  |  | F4 | 0.029 | 0.009 | 98.2 | 90.4 |
|  |  | F5 | 0.012 | 0.017 | 97.5 | 90.2 |
|  |  | F6 | 0.014 | 0.013 | 120.8 | 98.3 |
|  |  | F7 | −0.004 | −0.002 | 96.6 | 91.9 |
|  |  | F8 | 0.015 | −0.003 | 101.4 | 108.5 |
|  |  | F9 | 0.007 | 0.011 | 102.1 | 107.7 |
|  |  | F10 | −0.001 | 0.001 | 106.5 | 101.5 |
|  |  | F11 | 0.01 | −0.018 | 95.8 | 95.4 |
|  | **4.6** | F2 | 0.032 | −0.018 | 103.9 | 91.6 |
|  |  | F3 | 0.022 | 0.027 | 98.1 | 92.3 |
|  |  | F4 | 0.025 | 0.019 | 101.5 | 90.5 |
|  |  | F5 | 0.03 | 0.006 | 102.4 | 98.1 |
|  |  | F6 | 0.019 | 0.01 | 96.1 | 99.0 |
|  |  | F7 | −0.018 | 0.028 | 102.5 | 89.5 |
|  |  | F8 | 0.007 | 0.013 | 104.1 | 103.8 |
|  |  | F9 | 0.008 | 0.03 | 105.5 | 104.5 |
|  |  | F10 | −0.006 | 0.008 | 109.4 | 97.0 |
|  |  | F11 | 0.004 | 0.021 | 106.6 | 92.3 |
|  | **2.2** | F2 | 0.029 | 0.025 | 100.4 | 90.2 |
|  |  | F3 | 0.026 | 0.01 | 102.4 | 99.2 |
|  |  | F4 | −0.004 | 0.035 | 102.2 | 93.8 |
|  |  | F5 | 0.015 | −0.011 | 97.6 | 95.4 |
|  |  | F6 | −0.021 | 0.04 | 106.8 | 94.1 |
|  |  | F7 | 0.013 | 0.008 | 93.1 | 96.9 |
|  |  | F8 | −0.006 | 0 | 97.8 | 99.8 |
|  |  | F9 | 0.005 | −0.001 | 93.5 | 90.5 |
|  |  | F10 | −0.001 | −0.01 | 97.5 | 86.2 |
|  |  | F11 | −0.018 | 0.006 | 107.2 | 91.2 |

**Table 3.** HRK-blue hTLR2 Pam2CGDPKHPKSF (FSL-1) screening scores for 12 fruits. Quanti-blue (QB) score represents [Solvent control QB score] − [Fraction QB score]. The monosodium salt (WST-1) score represents [WST-1 score for each sample/WST-1 score for the media control (no ligand) *] × 100 which is a percentage.

| **Fruit** | **Concentration (mg/mL)** | **Fraction** | **QB  (Replicate 1)** | **QB  (Replicate 2)** | **WST-1 (%) (Replicate1)** | **WST-1 (%) (Replicate 2)** |
| --- | --- | --- | --- | --- | --- | --- |
| **Feijoa** | **17.8** | F2 | 0.029 | 0.061 | 93.9 | 89.9 |
|  |  | F3 | 0.047 | 0.069 | 102.4 | 98.4 |
|  |  | F4 | 0.06 | 0.042 | 105.0 | 104.3 |
|  |  | F5 | 0.014 | 0.039 | 106.1 | 98.8 |
|  |  | F6 | 0.001 | 0.037 | 112.6 | 110.3 |
|  |  | F7 | 0.079 | 0.008 | 103.4 | 100.0 |
|  |  | F8 | 0.083 | −0.04 | 114.8 | 108.7 |
|  |  | F9 | 0.051 | −0.04 | 114.7 | 116.1 |
|  |  | F10 | 0.054 | −0.023 | 106.4 | 117.7 |
|  |  | F11 | 0 | 0.001 | 107.6 | 113.1 |
|  | **8.9** | F2 | 0.038 | 0.01 | 84.0 | 97.1 |
|  |  | F3 | 0.073 | −0.001 | 96.3 | 100.4 |
|  |  | F4 | 0.081 | −0.004 | 100.3 | 109.1 |
|  |  | F5 | 0.054 | −0.01 | 104.0 | 112.3 |
|  |  | F6 | 0.065 | 0.049 | 104.6 | 100.4 |
|  |  | F7 | −0.022 | −0.007 | 99.6 | 101.7 |
|  |  | F8 | 0.034 | −0.002 | 100.4 | 101.7 |
|  |  | F9 | 0.03 | 0.023 | 94.5 | 112.1 |
|  |  | F10 | 0.024 | 0.001 | 107.3 | 121.5 |
|  |  | F11 | −0.023 | 0.033 | 92.6 | 114.0 |
|  | **4.6** | F2 | 0.017 | −0.01 | 101.8 | 101.4 |
|  |  | F3 | 0.066 | −0.003 | 105.3 | 101.5 |
|  |  | F4 | 0.045 | 0.011 | 106.4 | 100.0 |
|  |  | F5 | 0.006 | −0.019 | 106.1 | 108.4 |
|  |  | F6 | −0.002 | 0.028 | 108.6 | 100.8 |
|  |  | F7 | 0.08 | 0.022 | 101.9 | 101.4 |
|  |  | F8 | 0.036 | 0.018 | 103.8 | 103.6 |
|  |  | F9 | 0.046 | 0.034 | 104.1 | 110.0 |
|  |  | F10 | 0.023 | −0.005 | 104.7 | 108.6 |
|  |  | F11 | −0.009 | 0.033 | 98.7 | 116.9 |
|  | **2.2** | F2 | 0.012 | 0.012 | 94.1 | 101.7 |
|  |  | F3 | 0.052 | 0.009 | 103.3 | 99.6 |
|  |  | F4 | 0.02 | 0.005 | 111.0 | 107.6 |
|  |  | F5 | 0.019 | 0.027 | 105.7 | 105.8 |
|  |  | F6 | 0.009 | 0.029 | 108.8 | 104.6 |
|  |  | F7 | −0.004 | −0.009 | 94.0 | 101.2 |
|  |  | F8 | 0.004 | −0.012 | 99.2 | 106.7 |
|  |  | F9 | 0.041 | −0.022 | 96.0 | 103.7 |
|  |  | F10 | 0.052 | −0.032 | 98.8 | 104.5 |
|  |  | F11 | 0.02 | −0.01 | 102.9 | 102.4 |

**Table S3.** *Cont.*

| **Blackberry** | **17.8** | F2 | 0.018 | 0.019 | 104.5 | 97.8 |
| --- | --- | --- | --- | --- | --- | --- |
|  |  | F3 | 0.034 | 0.005 | 106.5 | 105.9 |
|  |  | F4 | 0.038 | 0.061 | 110.8 | 108.4 |
|  |  | F5 | 0.05 | 0.023 | 111.6 | 111.2 |
|  |  | F6 | 0.034 | 0.008 | 116.8 | 112.4 |
|  |  | F7 | 0.022 | 0.013 | 100.0 | 102.0 |
|  |  | F8 | 0.059 | −0.031 | 101.6 | 104.2 |
|  |  | F9 | 0.028 | 0.013 | 103.9 | 109.4 |
|  |  | F10 | 0.031 | −0.039 | 99.6 | 110.9 |
|  |  | F11 | 0.092 | 0.01 | 102.5 | 111.1 |
|  | **8.9** | F2 | 0.091 | −0.013 | 93.9 | 103.4 |
|  |  | F3 | 0.077 | 0.001 | 108.0 | 109.3 |
|  |  | F4 | 0.132 | 0.036 | 102.6 | 110.0 |
|  |  | F5 | 0.081 | 0.074 | 112.2 | 98.4 |
|  |  | F6 | 0.065 | −0.013 | 113.7 | 117.0 |
|  |  | F7 | −0.028 | 0.01 | 105.2 | 101.3 |
|  |  | F8 | −0.019 | 0.008 | 103.9 | 101.8 |
|  |  | F9 | −0.006 | 0.004 | 104.6 | 110.9 |
|  |  | F10 | −0.016 | 0.002 | 105.9 | 109.9 |
|  |  | F11 | −0.066 | −0.05 | 105.0 | 112.2 |
|  | **4.6** | F2 | 0.03 | −0.009 | 100.9 | 102.6 |
|  |  | F3 | 0.023 | −0.018 | 103.8 | 107.5 |
|  |  | F4 | 0.032 | 0.044 | 106.5 | 111.3 |
|  |  | F5 | 0.012 | 0.02 | 99.5 | 117.1 |
|  |  | F6 | 0.034 | 0.003 | 115.3 | 116.5 |
|  |  | F7 | 0.025 | 0.032 | 103.3 | 105.5 |
|  |  | F8 | 0.025 | 0.051 | 103.9 | 103.5 |
|  |  | F9 | −0.003 | 0.044 | 106.2 | 107.1 |
|  |  | F10 | 0.006 | 0.034 | 105.6 | 106.4 |
|  |  | F11 | −0.003 | 0.021 | 100.1 | 109.2 |
|  | **2.2** | F2 | 0 | 0.033 | 97.2 | 103.6 |
|  |  | F3 | 0.004 | 0.006 | 91.3 | 104.3 |
|  |  | F4 | 0.025 | 0.08 | 105.6 | 112.7 |
|  |  | F5 | 0.004 | 0.061 | 106.3 | 111.6 |
|  |  | F6 | −0.002 | 0.053 | 107.1 | 115.2 |
|  |  | F7 | −0.004 | 0.024 | 99.1 | 98.3 |
|  |  | F8 | 0.001 | −0.008 | 103.7 | 103.4 |
|  |  | F9 | 0.02 | 0.021 | 99.5 | 103.7 |
|  |  | F10 | 0.033 | −0.032 | 103.5 | 99.2 |
|  |  | F11 | 0.022 | −0.003 | 102.3 | 103.5 |

**Table S3.** *Cont.*

| **Elderberry** | **17.8** | F2 | 0.027 | −0.009 | 98.1 | 109.8 |
| --- | --- | --- | --- | --- | --- | --- |
|  |  | F3 | −0.001 | 0.018 | 101.8 | 102.6 |
|  |  | F4 | −0.008 | 0.012 | 99.9 | 102.5 |
|  |  | F5 | −0.012 | 0.002 | 100.6 | 104.4 |
|  |  | F6 | −0.003 | 0.002 | 100.1 | 113.4 |
|  |  | F7 | 0.001 | −0.007 | 90.8 | 100.1 |
|  |  | F8 | −0.002 | 0.009 | 96.6 | 93.7 |
|  |  | F9 | 0.019 | 0.002 | 94.1 | 99.7 |
|  |  | F10 | 0.022 | 0.012 | 99.9 | 99.0 |
|  |  | F11 | −0.022 | 0.001 | 90.4 | 106.1 |
|  | **8.9** | F2 | 0.027 | 0.022 | 93.9 | 105.1 |
|  |  | F3 | 0.009 | −0.002 | 95.6 | 103.8 |
|  |  | F4 | 0.008 | 0.013 | 102.1 | 99.7 |
|  |  | F5 | 0.002 | −0.02 | 102.9 | 94.9 |
|  |  | F6 | 0.016 | −0.007 | 107.5 | 110.3 |
|  |  | F7 | −0.015 | 0 | 103.6 | 103.5 |
|  |  | F8 | 0.06 | 0.013 | 102.1 | 109.0 |
|  |  | F9 | 0.017 | 0.015 | 105.2 | 100.0 |
|  |  | F10 | 0.01 | 0.015 | 103.5 | 114.6 |
|  |  | F11 | 0.022 | 0.015 | 100.2 | 123.3 |
|  | **4.6** | F2 | 0.025 | −0.038 | 86.8 | 102.2 |
|  |  | F3 | 0.006 | −0.009 | 91.4 | 95.6 |
|  |  | F4 | −0.034 | −0.004 | 94.2 | 99.0 |
|  |  | F5 | 0.011 | −0.018 | 89.5 | 102.5 |
|  |  | F6 | −0.033 | −0.044 | 97.8 | 105.2 |
|  |  | F7 | 0.023 | 0.052 | 94.8 | 101.0 |
|  |  | F8 | 0.06 | 0.04 | 92.1 | 102.3 |
|  |  | F9 | 0.069 | 0.063 | 96.4 | 94.2 |
|  |  | F10 | 0.063 | 0.05 | 99.2 | 104.8 |
|  |  | F11 | 0.049 | 0.062 | 99.8 | 110.5 |
|  | **2.2** | F2 | 0.017 | 0.036 | 95.2 | 115.2 |
|  |  | F3 | −0.014 | 0.052 | 101.2 | 95.5 |
|  |  | F4 | 0.036 | 0.018 | 99.2 | 103.6 |
|  |  | F5 | 0.023 | 0.045 | 95.5 | 105.8 |
|  |  | F6 | 0.042 | −0.015 | 96.6 | 101.3 |
|  |  | F7 | 0.007 | 0.012 | 99.8 | 98.7 |
|  |  | F8 | 0.07 | 0.021 | 96.2 | 95.5 |
|  |  | F9 | 0.088 | −0.002 | 97.6 | 103.9 |
|  |  | F10 | 0.079 | 0.025 | 92.1 | 98.3 |
|  |  | F11 | 0.011 | 0.002 | 96.6 | 106.0 |

**Table S3.** *Cont.*

| **Strawberry** | **17.8** | F2 | 0.023 | 0.015 | 89.9 | 95.4 |
| --- | --- | --- | --- | --- | --- | --- |
|  |  | F3 | −0.004 | 0.029 | 94.4 | 93.9 |
|  |  | F4 | 0.03 | 0.052 | 93.3 | 105.7 |
|  |  | F5 | −0.016 | 0.039 | 95.6 | 108.7 |
|  |  | F6 | −0.011 | 0.03 | 101.4 | 108.5 |
|  |  | F7 | 0.084 | 0.026 | 101.7 | 106.9 |
|  |  | F8 | 0.013 | 0.008 | 92.5 | 90.1 |
|  |  | F9 | −0.008 | −0.011 | 97.3 | 93.7 |
|  |  | F10 | 0.005 | −0.023 | 103.7 | 93.3 |
|  |  | F11 | −0.008 | −0.008 | 107.9 | 97.3 |
|  | **8.9** | F2 | −0.026 | −0.013 | 98.6 | 97.6 |
|  |  | F3 | 0.01 | −0.01 | 96.2 | 100.3 |
|  |  | F4 | 0.01 | 0.007 | 105.9 | 100.8 |
|  |  | F5 | 0.006 | 0.019 | 93.1 | 111.1 |
|  |  | F6 | −0.004 | 0.006 | 108.1 | 109.5 |
|  |  | F7 | 0.042 | 0.027 | 99.0 | 108.1 |
|  |  | F8 | 0.005 | 0.063 | 98.3 | 109.7 |
|  |  | F9 | −0.004 | 0.043 | 101.1 | 93.7 |
|  |  | F10 | −0.008 | 0.082 | 115.9 | 104.5 |
|  |  | F11 | −0.031 | 0.077 | 111.6 | 100.7 |
|  | **4.6** | F2 | 0 | 0 | 88.1 | 100.6 |
|  |  | F3 | 0.008 | −0.004 | 86.3 | 101.4 |
|  |  | F4 | −0.005 | −0.006 | 104.0 | 105.7 |
|  |  | F5 | −0.018 | −0.026 | 96.2 | 109.4 |
|  |  | F6 | −0.053 | −0.028 | 97.1 | 104.7 |
|  |  | F7 | −0.02 | 0.051 | 93.8 | 95.5 |
|  |  | F8 | 0.01 | 0.068 | 90.8 | 99.1 |
|  |  | F9 | −0.018 | 0.061 | 93.9 | 97.6 |
|  |  | F10 | −0.012 | 0.072 | 104.8 | 103.5 |
|  |  | F11 | −0.007 | 0.05 | 112.3 | 107.5 |
|  | **2.2** | F2 | −0.022 | −0.016 | 91.2 | 98.1 |
|  |  | F3 | −0.034 | −0.012 | 94.8 | 98.5 |
|  |  | F4 | 0.004 | −0.027 | 95.6 | 104.8 |
|  |  | F5 | −0.004 | −0.015 | 98.3 | 106.4 |
|  |  | F6 | −0.02 | −0.083 | 101.0 | 108.7 |
|  |  | F7 | 0.026 | −0.027 | 89.1 | 100.1 |
|  |  | F8 | 0.007 | 0.031 | 89.4 | 98.6 |
|  |  | F9 | 0.021 | 0.034 | 98.7 | 96.9 |
|  |  | F10 | 0.002 | 0.027 | 101.6 | 104.2 |
|  |  | F11 | 0.013 | 0.083 | 105.2 | 106.3 |

**Table S3.** *Cont.*

| **Cranberry** | **17.8** | F2 | −0.004 | −0.032 | 107.4 | 108.7 |
| --- | --- | --- | --- | --- | --- | --- |
|  |  | F3 | −0.023 | −0.041 | 100.4 | 108.2 |
|  |  | F4 | 0 | −0.019 | 103.0 | 109.8 |
|  |  | F5 | −0.02 | −0.02 | 107.3 | 103.0 |
|  |  | F6 | 0.005 | −0.03 | 109.9 | 109.2 |
|  |  | F7 | 0.028 | −0.026 | 114.1 | 111.4 |
|  |  | F8 | 0.005 | 0.016 | 101.6 | 105.9 |
|  |  | F9 | 0.026 | 0.022 | 99.4 | 101.3 |
|  |  | F10 | −0.009 | 0.055 | 103.3 | 102.9 |
|  |  | F11 | 0.015 | 0.034 | 101.5 | 97.5 |
|  | **8.9** | F2 | 0.042 | 0.009 | 100.8 | 116.1 |
|  |  | F3 | −0.005 | −0.021 | 95.7 | 97.1 |
|  |  | F4 | −0.001 | −0.032 | 95.9 | 103.5 |
|  |  | F5 | 0.002 | −0.012 | 110.7 | 113.2 |
|  |  | F6 | 0.005 | −0.01 | 108.5 | 101.9 |
|  |  | F7 | 0.021 | 0.033 | 113.0 | 107.3 |
|  |  | F8 | 0.018 | 0.044 | 102.0 | 108.1 |
|  |  | F9 | 0.018 | 0.061 | 101.1 | 117.9 |
|  |  | F10 | 0.016 | 0.018 | 108.4 | 106.5 |
|  |  | F11 | 0.002 | 0.075 | 105.5 | 101.8 |
|  | **4.6** | F2 | 0.042 | 0.022 | 107.9 | 120.2 |
|  |  | F3 | 0.01 | 0.001 | 88.7 | 111.4 |
|  |  | F4 | 0.005 | −0.027 | 100.1 | 101.0 |
|  |  | F5 | 0.035 | −0.024 | 104.3 | 105.4 |
|  |  | F6 | 0.02 | −0.002 | 101.7 | 98.6 |
|  |  | F7 | 0.019 | 0.021 | 114.7 | 112.5 |
|  |  | F8 | 0.033 | −0.004 | 107.8 | 119.4 |
|  |  | F9 | 0.024 | −0.001 | 98.2 | 106.0 |
|  |  | F10 | −0.005 | 0.005 | 107.3 | 124.1 |
|  |  | F11 | 0.018 | −0.018 | 100.8 | 99.0 |
|  | **2.2** | F2 | 0.037 | −0.001 | 104.2 | 111.7 |
|  |  | F3 | −0.01 | 0.012 | 97.9 | 116.6 |
|  |  | F4 | −0.01 | −0.007 | 98.8 | 103.1 |
|  |  | F5 | 0.007 | −0.031 | 99.9 | 112.0 |
|  |  | F6 | −0.02 | −0.039 | 102.4 | 108.6 |
|  |  | F7 | 0.027 | 0.007 | 120.7 | 123.0 |
|  |  | F8 | 0.004 | 0.022 | 115.6 | 106.4 |
|  |  | F9 | −0.001 | −0.013 | 100.6 | 118.4 |
|  |  | F10 | −0.03 | −0.007 | 104.0 | 102.8 |
|  |  | F11 | −0.008 | 0.037 | 102.9 | 106.2 |

**Table S3.** *Cont.*

| **Red Raspberry** | **17.8** | F2 | 0.041 | −0.026 | 93.4 | 99.0 |
| --- | --- | --- | --- | --- | --- | --- |
|  |  | F3 | 0.006 | −0.011 | 127.5 | 96.6 |
|  |  | F4 | 0.057 | −0.007 | 106.6 | 78.4 |
|  |  | F5 | 0.061 | 0.015 | 99.3 | 103.4 |
|  |  | F6 | 0.074 | −0.042 | 102.6 | 97.7 |
|  |  | F7 | 0.032 | −0.01 | 74.8 | 79.7 |
|  |  | F8 | 0.061 | 0.002 | 73.3 | 72.0 |
|  |  | F9 | 0.02 | −0.04 | 72.1 | 77.6 |
|  |  | F10 | −0.004 | −0.04 | 76.7 | 74.7 |
|  |  | F11 | −0.023 | −0.045 | 78.1 | 82.1 |
|  | **8.9** | F2 | 0.023 | 0.05 | 79.8 | 138.0 |
|  |  | F3 | 0.024 | 0.027 | 73.3 | 102.6 |
|  |  | F4 | 0.062 | 0.062 | 86.8 | 84.6 |
|  |  | F5 | 0.056 | 0.054 | 82.1 | 81.9 |
|  |  | F6 | 0.055 | 0.034 | 93.1 | 107.8 |
|  |  | F7 | 0.01 | 0.004 | 88.7 | 76.6 |
|  |  | F8 | 0.042 | −0.01 | 86.3 | 69.7 |
|  |  | F9 | 0.017 | 0.033 | 100.0 | 81.5 |
|  |  | F10 | 0.005 | −0.011 | 86.0 | 78.4 |
|  |  | F11 | 0 | 0.001 | 82.9 | 74.6 |
|  | **4.6** | F2 | 0.002 | 0.039 | 101.7 | 83.3 |
|  |  | F3 | 0.05 | 0.023 | 125.9 | 88.5 |
|  |  | F4 | 0.011 | 0.003 | 114.2 | 77.4 |
|  |  | F5 | 0.001 | 0.023 | 106.1 | 79.5 |
|  |  | F6 | −0.002 | 0.027 | 84.9 | 83.5 |
|  |  | F7 | 0.012 | −0.003 | 89.7 | 84.3 |
|  |  | F8 | −0.009 | −0.011 | 89.6 | 75.0 |
|  |  | F9 | 0.017 | 0.014 | 84.6 | 70.4 |
|  |  | F10 | −0.018 | −0.028 | 74.1 | 73.6 |
|  |  | F11 | −0.023 | −0.025 | 76.9 | 90.9 |
|  | **2.2** | F2 | 0.007 | −0.011 | 85.0 | 91.4 |
|  |  | F3 | −0.006 | −0.018 | 70.5 | 86.7 |
|  |  | F4 | 0.022 | −0.017 | 105.1 | 77.6 |
|  |  | F5 | 0.01 | −0.023 | 84.3 | 86.8 |
|  |  | F6 | 0.019 | −0.028 | 79.6 | 113.9 |
|  |  | F7 | 0.017 | 0.041 | 71.3 | 72.7 |
|  |  | F8 | 0.001 | 0.042 | 90.1 | 66.7 |
|  |  | F9 | 0.006 | 0.011 | 73.0 | 72.9 |
|  |  | F10 | 0.001 | 0.009 | 75.8 | 78.4 |
|  |  | F11 | 0.002 | 0.016 | 82.4 | 87.5 |

**Table S3.** *Cont.*

| **Blackcurrant** | **17.8** | F2 | 0 | 0.025 | 106.1 | 106.7 |
| --- | --- | --- | --- | --- | --- | --- |
|  |  | F3 | −0.01 | 0.013 | 117.6 | 98.1 |
|  |  | F4 | 0.011 | 0.005 | 82.9 | 162.8 |
|  |  | F5 | 0.012 | 0.028 | 83.9 | 148.6 |
|  |  | F6 | −0.019 | −0.009 | 96.1 | 100.5 |
|  |  | F7 | 0.029 | 0.005 | 108.2 | 112.7 |
|  |  | F8 | 0.024 | −0.044 | 116.5 | 82.5 |
|  |  | F9 | −0.004 | −0.02 | 166.4 | 82.5 |
|  |  | F10 | 0.039 | −0.029 | 95.6 | 116.8 |
|  |  | F11 | −0.005 | −0.007 | 90.0 | 104.2 |
|  | **8.9** | F2 | 0.03 | 0.003 | 109.6 | 134.0 |
|  |  | F3 | −0.012 | −0.008 | 116.8 | 87.8 |
|  |  | F4 | −0.013 | −0.02 | 97.3 | 133.0 |
|  |  | F5 | 0.012 | −0.033 | 96.7 | 115.0 |
|  |  | F6 | −0.003 | −0.033 | 101.6 | 90.4 |
|  |  | F7 | 0.043 | 0.019 | 105.4 | 85.3 |
|  |  | F8 | 0.004 | −0.024 | 121.7 | 82.1 |
|  |  | F9 | 0.004 | 0.001 | 98.5 | 86.5 |
|  |  | F10 | 0.023 | −0.021 | 132.8 | 83.0 |
|  |  | F11 | 0.002 | −0.016 | 99.5 | 94.5 |
|  | **4.6** | F2 | −0.032 | −0.032 | 104.6 | 96.3 |
|  |  | F3 | −0.034 | −0.03 | 89.0 | 90.0 |
|  |  | F4 | −0.018 | −0.035 | 91.8 | 145.1 |
|  |  | F5 | −0.014 | −0.067 | 114.8 | 93.0 |
|  |  | F6 | −0.038 | −0.079 | 99.5 | 95.3 |
|  |  | F7 | 0.018 | −0.007 | 98.1 | 131.5 |
|  |  | F8 | 0.018 | −0.013 | 86.4 | 79.4 |
|  |  | F9 | 0.003 | −0.026 | 86.2 | 83.9 |
|  |  | F10 | −0.004 | 0.024 | 92.1 | 81.3 |
|  |  | F11 | 0.017 | −0.042 | 104.4 | 98.5 |
|  | **2.2** | F2 | −0.016 | −0.04 | 117.2 | 118.3 |
|  |  | F3 | −0.022 | −0.01 | 129.2 | 95.7 |
|  |  | F4 | −0.041 | −0.057 | 91.8 | 136.9 |
|  |  | F5 | 0.001 | −0.008 | 152.4 | 122.0 |
|  |  | F6 | −0.029 | −0.066 | 100.6 | 96.3 |
|  |  | F7 | 0.011 | 0.007 | 102.5 | 110.4 |
|  |  | F8 | 0.009 | −0.017 | 89.8 | 83.8 |
|  |  | F9 | −0.014 | −0.016 | 84.9 | 93.9 |
|  |  | F10 | 0.017 | −0.005 | 95.7 | 111.4 |
|  |  | F11 | −0.025 | −0.047 | 108.4 | 95.3 |

**Table S3.** *Cont.*

| **Green Grapes** | **17.8** | F2 | 0.016 | 0.005 | 94.0 | 97.2 |
| --- | --- | --- | --- | --- | --- | --- |
|  |  | F3 | 0.039 | 0.016 | 97.6 | 91.0 |
|  |  | F4 | 0.015 | 0.007 | 101.7 | 92.0 |
|  |  | F5 | 0.002 | 0.008 | 101.5 | 98.2 |
|  |  | F6 | −0.021 | −0.001 | 103.1 | 93.0 |
|  |  | F7 | 0.011 | −0.004 | 106.0 | 106.2 |
|  |  | F8 | 0.047 | 0.007 | 102.8 | 105.2 |
|  |  | F9 | 0.003 | 0.006 | 110.1 | 101.4 |
|  |  | F10 | 0.013 | −0.016 | 111.7 | 105.0 |
|  |  | F11 | 0.012 | −0.001 | 102.4 | 93.8 |
|  | **8.9** | F2 | 0.003 | 0.007 | 104.7 | 98.6 |
|  |  | F3 | 0.043 | 0.026 | 108.3 | 100.6 |
|  |  | F4 | −0.002 | 0.003 | 113.3 | 94.8 |
|  |  | F5 | 0.013 | 0.027 | 115.4 | 91.6 |
|  |  | F6 | −0.027 | 0.011 | 105.8 | 98.0 |
|  |  | F7 | 0 | 0.001 | 102.6 | 90.8 |
|  |  | F8 | 0.023 | −0.006 | 99.9 | 94.2 |
|  |  | F9 | 0.031 | 0.003 | 103.5 | 85.2 |
|  |  | F10 | 0.03 | 0 | 99.7 | 97.4 |
|  |  | F11 | 0.035 | 0.004 | 104.2 | 86.6 |
|  | **4.6** | F2 | −0.013 | 0.038 | 105.6 | 94.4 |
|  |  | F3 | 0.008 | 0.03 | 112.9 | 94.6 |
|  |  | F4 | −0.009 | 0.006 | 113.5 | 95.8 |
|  |  | F5 | −0.043 | 0.03 | 115.4 | 98.2 |
|  |  | F6 | −0.028 | 0.035 | 110.8 | 90.4 |
|  |  | F7 | 0.017 | 0.036 | 105.1 | 110.6 |
|  |  | F8 | −0.016 | 0.039 | 114.9 | 105.2 |
|  |  | F9 | 0.007 | 0.044 | 108.5 | 96.0 |
|  |  | F10 | 0.009 | 0.051 | 110.8 | 89.0 |
|  |  | F11 | 0.036 | 0.026 | 106.0 | 87.0 |
|  | **2.2** | F2 | 0.028 | 0.021 | 107.2 | 110.0 |
|  |  | F3 | 0.056 | 0.001 | 111.5 | 108.8 |
|  |  | F4 | 0.044 | −0.002 | 116.0 | 105.6 |
|  |  | F5 | 0.056 | 0.016 | 110.8 | 102.6 |
|  |  | F6 | 0.034 | 0.027 | 104.2 | 97.6 |
|  |  | F7 | 0.003 | 0.011 | 104.2 | 106.8 |
|  |  | F8 | −0.001 | 0.012 | 105.6 | 97.8 |
|  |  | F9 | 0.005 | 0.018 | 105.8 | 99.4 |
|  |  | F10 | −0.002 | 0.024 | 107.2 | 93.2 |
|  |  | F11 | −0.007 | 0.013 | 110.6 | 96.2 |

**Table S3.** *Cont.*

| **Black Grapes** | **17.8** | F2 | 0.001 | 0.005 | 92.8 | 120.2 |
| --- | --- | --- | --- | --- | --- | --- |
|  |  | F3 | −0.019 | 0.006 | 100.3 | 114.6 |
|  |  | F4 | 0.026 | 0.004 | 98.1 | 109.9 |
|  |  | F5 | 0.004 | 0.013 | 101.6 | 116.0 |
|  |  | F6 | 0.011 | 0.007 | 106.0 | 120.2 |
|  |  | F7 | −0.007 | 0.029 | 100.3 | 117.4 |
|  |  | F8 | −0.017 | 0.017 | 103.4 | 117.9 |
|  |  | F9 | 0 | 0.058 | 101.6 | 105.0 |
|  |  | F10 | 0.037 | 0.036 | 103.8 | 120.2 |
|  |  | F11 | 0.005 | 0.003 | 101.2 | 110.2 |
|  | **8.9** | F2 | 0.061 | −0.019 | 97.2 | 104.7 |
|  |  | F3 | 0.021 | −0.028 | 98.4 | 113.0 |
|  |  | F4 | 0.025 | −0.043 | 96.8 | 103.6 |
|  |  | F5 | 0.037 | −0.029 | 95.5 | 119.1 |
|  |  | F6 | 0.02 | 0 | 105.1 | 117.6 |
|  |  | F7 | −0.003 | 0.022 | 103.2 | 128.4 |
|  |  | F8 | 0.021 | 0.029 | 90.2 | 123.0 |
|  |  | F9 | 0.012 | 0.009 | 92.4 | 105.6 |
|  |  | F10 | 0.061 | 0.046 | 95.1 | 110.9 |
|  |  | F11 | 0.004 | 0.05 | 100.8 | 113.9 |
|  | **4.6** | F2 | 0.052 | −0.011 | 101.4 | 123.9 |
|  |  | F3 | 0.033 | −0.029 | 98.5 | 123.2 |
|  |  | F4 | 0.043 | −0.02 | 93.3 | 110.6 |
|  |  | F5 | 0.016 | −0.015 | 99.4 | 122.8 |
|  |  | F6 | −0.001 | −0.012 | 105.9 | 110.7 |
|  |  | F7 | 0.005 | 0.021 | 106.8 | 118.5 |
|  |  | F8 | −0.006 | −0.013 | 111.2 | 119.6 |
|  |  | F9 | 0.018 | 0.014 | 101.8 | 129.9 |
|  |  | F10 | 0.013 | 0.022 | 115.1 | 111.4 |
|  |  | F11 | 0.001 | 0.039 | 109.5 | 124.8 |
|  | **2.2** | F2 | −0.007 | 0.007 | 111.0 | 126.7 |
|  |  | F3 | −0.01 | −0.016 | 100.7 | 117.8 |
|  |  | F4 | −0.011 | −0.008 | 102.7 | 121.5 |
|  |  | F5 | −0.019 | −0.007 | 106.6 | 118.0 |
|  |  | F6 | −0.014 | −0.047 | 101.1 | 117.5 |
|  |  | F7 | −0.001 | 0.015 | 103.2 | 115.8 |
|  |  | F8 | 0.001 | −0.019 | 110.1 | 115.9 |
|  |  | F9 | 0.017 | −0.006 | 105.5 | 97.0 |
|  |  | F10 | −0.001 | −0.008 | 106.7 | 102.4 |
|  |  | F11 | −0.012 | 0.011 | 108.2 | 114.1 |

**Table S3.** *Cont.*

| **Pear** | **17.8** | F2 | −0.02 | −0.013 | 104.4 | 113.1 |
| --- | --- | --- | --- | --- | --- | --- |
|  |  | F3 | −0.007 | −0.004 | 105.8 | 101.8 |
|  |  | F4 | 0.009 | −0.001 | 102.9 | 107.7 |
|  |  | F5 | 0.018 | 0.007 | 113.3 | 115.1 |
|  |  | F6 | 0.005 | −0.051 | 114.6 | 114.4 |
|  |  | F7 | 0.013 | −0.001 | 96.2 | 125.4 |
|  |  | F8 | −0.016 | −0.014 | 109.5 | 123.0 |
|  |  | F9 | 0.018 | −0.008 | 114.4 | 112.9 |
|  |  | F10 | −0.04 | −0.045 | 111.3 | 113.9 |
|  |  | F11 | −0.03 | −0.032 | 115.4 | 116.4 |
|  | **8.9** | F2 | 0.008 | 0.011 | 104.3 | 112.5 |
|  |  | F3 | 0.005 | −0.01 | 95.2 | 102.1 |
|  |  | F4 | −0.01 | −0.087 | 94.5 | 106.8 |
|  |  | F5 | −0.003 | −0.009 | 105.0 | 112.7 |
|  |  | F6 | −0.02 | −0.079 | 109.7 | 110.1 |
|  |  | F7 | −0.002 | 0.025 | 101.0 | 118.4 |
|  |  | F8 | 0.017 | 0.032 | 111.0 | 113.8 |
|  |  | F9 | −0.021 | −0.005 | 99.9 | 113.9 |
|  |  | F10 | −0.023 | 0.009 | 119.8 | 128.1 |
|  |  | F11 | −0.013 | −0.021 | 110.0 | 124.9 |
|  | **4.6** | F2 | 0.017 | 0.001 | 93.8 | 96.3 |
|  |  | F3 | −0.044 | −0.01 | 100.8 | 109.2 |
|  |  | F4 | 0.001 | −0.026 | 107.6 | 109.0 |
|  |  | F5 | −0.027 | −0.05 | 98.7 | 112.8 |
|  |  | F6 | −0.011 | −0.029 | 108.2 | 116.2 |
|  |  | F7 | 0.022 | 0.023 | 104.6 | 125.3 |
|  |  | F8 | 0.014 | 0.007 | 101.7 | 105.7 |
|  |  | F9 | 0.005 | −0.014 | 103.8 | 103.7 |
|  |  | F10 | 0.007 | 0.006 | 122.1 | 136.5 |
|  |  | F11 | −0.004 | −0.038 | 124.7 | 114.8 |
|  | **2.2** | F2 | 0.002 | 0.01 | 101.6 | 95.5 |
|  |  | F3 | −0.037 | −0.03 | 110.6 | 104.4 |
|  |  | F4 | −0.014 | −0.031 | 106.1 | 117.8 |
|  |  | F5 | −0.035 | −0.027 | 114.0 | 114.7 |
|  |  | F6 | −0.058 | −0.012 | 118.4 | 122.2 |
|  |  | F7 | −0.013 | 0.019 | 105.1 | 93.9 |
|  |  | F8 | −0.013 | 0.005 | 95.9 | 90.6 |
|  |  | F9 | −0.017 | 0.014 | 102.3 | 117.2 |
|  |  | F10 | −0.014 | 0.028 | 127.6 | 107.0 |
|  |  | F11 | −0.024 | −0.005 | 124.8 | 117.7 |

**Table S3.** *Cont.*

| **Mangosteen** | **17.8** | F2 | 0.005 | 0.003 | 106.4 | 108.9 |
| --- | --- | --- | --- | --- | --- | --- |
|  |  | F3 | 0 | 0.005 | 95.7 | 112.0 |
|  |  | F4 | −0.021 | 0.021 | 97.0 | 112.7 |
|  |  | F5 | 0.005 | 0.025 | 105.3 | 114.8 |
|  |  | F6 | −0.019 | 0.012 | 104.0 | 116.7 |
|  |  | F7 | 0.018 | −0.004 | 96.8 | 101.3 |
|  |  | F8 | −0.01 | −0.038 | 90.1 | 99.3 |
|  |  | F9 | 0.02 | −0.038 | 96.6 | 104.8 |
|  |  | F10 | 0.015 | −0.046 | 97.0 | 110.0 |
|  |  | F11 | −0.016 | −0.063 | 97.3 | 113.3 |
|  | **8.9** | F2 | −0.032 | 0.005 | 96.8 | 101.8 |
|  |  | F3 | 0.024 | −0.03 | 101.2 | 101.4 |
|  |  | F4 | −0.005 | −0.041 | 100.8 | 114.6 |
|  |  | F5 | −0.025 | −0.059 | 93.6 | 116.0 |
|  |  | F6 | 0.016 | −0.018 | 98.6 | 117.1 |
|  |  | F7 | 0 | 0.014 | 91.8 | 97.0 |
|  |  | F8 | −0.012 | −0.003 | 87.7 | 102.3 |
|  |  | F9 | −0.001 | −0.02 | 96.0 | 107.8 |
|  |  | F10 | −0.038 | −0.041 | 94.0 | 117.1 |
|  |  | F11 | −0.018 | −0.02 | 99.3 | 119.4 |
|  | **4.6** | F2 | 0.013 | 0.012 | 114.5 | 107.0 |
|  |  | F3 | 0.013 | −0.002 | 92.6 | 106.8 |
|  |  | F4 | 0.02 | −0.001 | 86.2 | 106.3 |
|  |  | F5 | 0.002 | −0.04 | 87.9 | 114.7 |
|  |  | F6 | 0.02 | −0.029 | 96.9 | 114.5 |
|  |  | F7 | 0.024 | 0.014 | 104.6 | 97.0 |
|  |  | F8 | −0.006 | −0.042 | 112.3 | 95.6 |
|  |  | F9 | 0.032 | −0.015 | 89.0 | 106.6 |
|  |  | F10 | −0.014 | −0.023 | 100.1 | 111.7 |
|  |  | F11 | 0.016 | −0.024 | 100.1 | 114.6 |
|  | **2.2** | F2 | 0.017 | −0.048 | 107.6 | 101.6 |
|  |  | F3 | −0.024 | −0.024 | 82.8 | 110.7 |
|  |  | F4 | −0.025 | −0.002 | 93.5 | 114.4 |
|  |  | F5 | 0 | −0.049 | 90.3 | 114.5 |
|  |  | F6 | 0.016 | −0.043 | 80.5 | 111.7 |
|  |  | F7 | −0.021 | 0.008 | 89.7 | 90.7 |
|  |  | F8 | −0.055 | −0.023 | 103.6 | 97.0 |
|  |  | F9 | −0.018 | −0.039 | 93.5 | 98.2 |
|  |  | F10 | −0.058 | −0.024 | 102.7 | 98.7 |
|  |  | F11 | −0.061 | −0.019 | 101.5 | 111.3 |

**Table S3.** *Cont.*

| **Plum** | **17.8** | F2 | 0.009 | 0.004 | 98.7 | 102.2 |
| --- | --- | --- | --- | --- | --- | --- |
|  |  | F3 | 0.004 | 0.003 | 89.1 | 100.6 |
|  |  | F4 | 0.03 | −0.007 | 87.3 | 99.6 |
|  |  | F5 | 0.018 | 0 | 89.1 | 90.9 |
|  |  | F6 | 0.022 | 0.02 | 104.5 | 95.4 |
|  |  | F7 | 0.001 | −0.015 | 129.8 | 105.8 |
|  |  | F8 | 0.047 | 0.004 | 97.3 | 102.3 |
|  |  | F9 | 0.025 | −0.021 | 92.7 | 93.4 |
|  |  | F10 | −0.014 | −0.059 | 97.1 | 98.1 |
|  |  | F11 | 0.004 | 0.01 | 106.7 | 103.3 |
|  | **8.9** | F2 | −0.015 | 0.008 | 118.3 | 91.1 |
|  |  | F3 | −0.026 | −0.047 | 114.7 | 85.3 |
|  |  | F4 | −0.05 | −0.041 | 107.6 | 86.9 |
|  |  | F5 | −0.056 | 0.009 | 104.7 | 88.5 |
|  |  | F6 | −0.01 | 0.019 | 121.8 | 97.4 |
|  |  | F7 | 0.012 | 0.007 | 121.8 | 113.3 |
|  |  | F8 | 0.017 | 0.007 | 96.8 | 96.4 |
|  |  | F9 | 0.023 | −0.008 | 93.3 | 96.7 |
|  |  | F10 | 0.003 | 0.015 | 100.7 | 93.1 |
|  |  | F11 | 0.01 | 0.001 | 104.1 | 98.3 |
|  | **4.6** | F2 | −0.003 | −0.014 | 120.6 | 115.0 |
|  |  | F3 | 0.016 | −0.018 | 114.6 | 104.2 |
|  |  | F4 | −0.001 | −0.031 | 106.6 | 101.4 |
|  |  | F5 | 0.007 | −0.039 | 108.1 | 96.7 |
|  |  | F6 | −0.007 | −0.066 | 110.7 | 92.9 |
|  |  | F7 | 0.015 | 0.035 | 119.5 | 109.5 |
|  |  | F8 | 0.038 | 0.005 | 104.5 | 98.6 |
|  |  | F9 | 0.044 | 0.004 | 108.1 | 96.7 |
|  |  | F10 | −0.001 | 0.048 | 105.6 | 92.9 |
|  |  | F11 | −0.002 | −0.015 | 109.9 | 92.2 |
|  | **2.2** | F2 | 0.005 | 0 | 119.4 | 112.8 |
|  |  | F3 | 0.009 | −0.008 | 96.8 | 101.9 |
|  |  | F4 | −0.005 | −0.003 | 100.2 | 95.1 |
|  |  | F5 | −0.043 | 0.02 | 100.5 | 95.5 |
|  |  | F6 | 0.021 | −0.008 | 126.0 | 99.7 |
|  |  | F7 | 0.003 | −0.042 | 123.4 | 107.1 |
|  |  | F8 | 0.008 | −0.027 | 104.4 | 106.2 |
|  |  | F9 | 0.002 | −0.028 | 110.9 | 90.1 |
|  |  | F10 | −0.016 | −0.033 | 108.2 | 92.7 |
|  |  | F11 | 0.02 | −0.047 | 104.9 | 92.5 |

© 2014 by the authors; licensee MDPI, Basel, Switzerland. This article is an open access article distributed under the terms and conditions of the Creative Commons Attribution license (http://creativecommons.org/licenses/by/4.0/).
